# Natural variation of an EF-hand Ca^2+^-binding-protein coding gene confers saline-alkaline tolerance in maize

**DOI:** 10.1038/s41467-019-14027-y

**Published:** 2020-01-10

**Authors:** Yibo Cao, Ming Zhang, Xiaoyan Liang, Fenrong Li, Yunlu Shi, Xiaohong Yang, Caifu Jiang

**Affiliations:** 1 State Key Laboratory of Plant Physiology and Biochemistry, College of Biological Sciences, China Agricultural University, Beijing, 100094 China; 2 Center for Crop Functional Genomics and Molecular Breeding, China Agricultural University, Beijing, 100094 China; 3 Laboratory of Agrobiotechnology and National Maize Improvement Center of China, MOA Key Lab of Maize Biology, China Agricultural University, Beijing, 100193 China; 4 Outstanding Discipline Program for the Universities in Beijing, Beijing, 100094 China

**Keywords:** Agricultural genetics, Agricultural genetics, Natural variation in plants, Salt

## Abstract

Sodium (Na^+^) toxicity is one of the major damages imposed on crops by saline-alkaline stress. Here we show that natural maize inbred lines display substantial variations in shoot Na^+^ contents and saline-alkaline (NaHCO_3_) tolerance, and reveal that *ZmNSA1* (*Na*^*+*^ *Content under Saline-Alkaline Condition*) confers shoot Na^+^ variations under NaHCO_3_ condition by a genome-wide association study. Lacking of ZmNSA1 promotes shoot Na^+^ homeostasis by increasing root Na^+^ efflux. A naturally occurred 4-bp deletion decreases the translation efficiency of *ZmNSA1* mRNA, thus promotes Na^+^ homeostasis. We further show that, under saline-alkaline condition, Ca^2+^ binds to the EF-hand domain of ZmNSA1 then triggers its degradation via 26S proteasome, which in turn increases the transcripts levels of PM-H^+^-ATPases (*MHA2* and *MHA4*), and consequently enhances SOS1 Na^+^/H^+^ antiporter-mediated root Na^+^ efflux. Our studies reveal the mechanism of Ca^2+^-triggered saline-alkaline tolerance and provide an important gene target for breeding saline-alkaline tolerant maize varieties.

## Abstract (web summary)

Saline-alkaline stress affects worldwide crops production, but the tolerance mechanisms have not been fully elucidated. Here, the authors show that EF-hand Ca2 + -binding-protein coding gene *ZmNSA1* can regulate root H + efflux, Na + homeostasis, and saline-alkaline tolerance in maize.

## Introduction

Saline-alkaline stress is a widely spread abiotic stress affecting an estimation of 4.15 × 10^8^ ha of lands all over the world^1^, which is emerging as a major constraint of global crop production^2^. Saline-alkaline soil is characterized by high salt (salinity) and high pH (above pH 8.0; alkalinity)^3^, which causes combined damages of high pH stress, ion toxicity, and osmotic stress^3–5^. In order to cope with saline-alkaline stress, plants have evolved a range of adaptive strategies. For instance, the H^+^ efflux from root to soil acidifies the rhizosphere then promotes adaptation to high pH stress^6^, the Na^+^-preferring transporters (e.g., SOS1 and HKT1) enable the circumvention of Na^+^ toxicity^2,7^, the accumulation of osmoprotectants (e.g., glycinebetaine) attenuates osmotic damage^8^. These adaptive mechanisms act together to enable plant to survive saline-alkaline stress. Up to this day, it remains largely unknown how plants sense saline-alkaline stress and convert it into second signaling messengers (e.g., Ca^2+^), and how plants decode the second messengers then activate/inactivate the downstream responses.

The major natural basic salts in saline-alkaline farmlands are sodium hydrogen carbonate (NaHCO_3_) and sodium carbonate (Na_2_CO_3_)^9^, and Na^+^ is the most abundance soluble salt in the saline-alkaline farmlands. Excessive accumulation of tissue Na^+^ is deleterious for most crops, thus the maintenance of Na^+^ homeostasis is essential for the crop saline-alkaline tolerance. Previous studies have shown that Na^+^ selective transporters substantially confer cellular and whole-plant Na^+^ homeostasis^2,7^. For example, SOS1 Na^+^/H^+^ antiporters (e.g., AtSOS1) transport Na^+^ out of root cells^7,10^, the HKT family Na^+^ transporters (e.g., AtHKT1) regulate long distance Na^+^ delivery, e.g., the root-to-shoot Na^+^ translocation and Na^+^ exclusion from the reproductive organ^11–16^. These Na^+^ transporters and their regulators (e.g., SOS2 and SOS3) act together to circumvent Na^+^ toxicity^2,7,17^. Under saline-alkaline condition, the increases of rhizosphere and cytosolic pH weaken the function of H^+^-gradient-dependent Na^+^ transporters (e.g., SOS1 Na^+^/H^+^ antiporters), then boosting Na^+^ damage^18^. Therefore, maintaining the H^+^ gradient across the plasma membrane is essential for Na^+^ homeostasis, especially under saline-alkaline conditions (see below).

The optimal cytoplasmic pH for plant cells is neutral pH. When the rhizosphere pH is lower than the pH in the cytosolic of root cells, the H^+^ gradient across the plasma membrane (membrane potential) drives uptake of nutrients (e.g., phosphorus, nitrate and iron)^19–21^. Under saline-alkaline condition, plants have to export cellular H^+^ to rhizosphere to maintain the membrane potential^22,23^, and the PM-H^+^-ATPase is the major pump mediating root H^+^ efflux^6^. Previous studies in *Arabidopsis* have shown that the activity of the PM-H^+^-ATPase AHA2 is inhibited by its C-terminal mediated auto-inhibition and by PKS5 mediated phosphorylation at Ser^931^. The saline-alkaline stress induces the increase of cytosolic Ca^2+^, which binds to the 14-3-3 proteins and triggers its interaction with PKS5, then inhibits PKS5 activity thus activates AHA2^6^. In the meantime, a phosphorylation at Thr^947^ activates AHA2 via triggers its interaction with the dimeric 14-3-3 proteins^6,18,24^. These posttranscriptional mechanisms act together to activate AHA2, then promotes root H^+^ efflux, thereby activating SOS1 Na^+^/H^+^ antiporter and other adaptive responses^18,25^. Moreover, previous studies have also suggested that the transcript levels of some PM-H^+^-ATPase increased under stress conditions, e.g., phosphorus deficiency increases the transcript levels of *AHA2* and *AHA7*^26^, iron deficiency increases the expression of *AHA2* and *AHA7*^27^, salt stress upregulates the transcript levels of *AHA2*^28^. These observations indicate that the transcriptional regulation of PM-H^+^-ATPase is also important for the regulation of root H^+^ efflux under stress conditions, nevertheless, the mechanism remains largely unknown.

Maize (*Zea mays ssp. mays*) is a glycophytic specie that is sensitive to saline-alkaline stress^29^. Previous studies have shown that natural maize inbred lines show large variations of sensitivity to saline and saline-alkaline stress, and which is substantially attributed to the variations of shoot Na^+^ contents^30,31^. Here, we show that a calcium-binding EF-hand protein ZmNSA1 underlies the natural variations of shoot-Na^+^ contents under NaHCO_3_ condition by a GWAS analysis. Lacking of ZmNSA1 increases root Na^+^ efflux, then promotes shoot Na^+^ exclusion and saline-alkaline tolerance. The functional variation of ZmNSA1 is ascribed to a 4-bp deletion located in the 3′UTR of *ZmNSA1*, which decreases the abundance of ZmNSA1 protein by reducing the translation efficiency of *ZmNSA1* mRNA. We further show that, under saline-alkaline condition, Ca^2+^ binds ZmNSA1 and triggers its degradation via 26S proteasome, then increases the expression of PM-H^+^-ATPases, thereby promoting root H^+^ efflux and SOS1 Na^+^/H^+^ antiporter-mediated root Na^+^ efflux, ultimately promoting saline-alkaline tolerance. Our study shows how Ca^2+^ triggered degradation of a Ca^2+^-binding EF-hand protein confers transcriptional upregulation of PM-H^+^-ATPases and saline-alkaline tolerance, providing a mechanistic understanding of crop saline-alkaline stress tolerance and an important genetic target for breeding saline-alkaline tolerant maize varieties.

## Results

### High pH stress disturbs Na^+^ homeostasis in maize

**Fig. 1:**
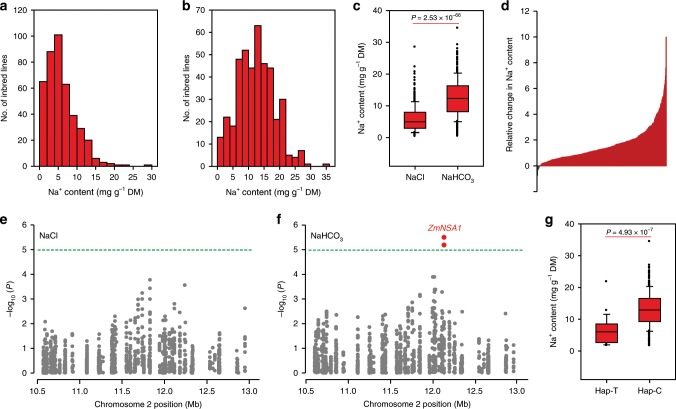
*ZmNSA1* confers natural variations of shoot Na^+^ contents under NaHCO_3_ condition. **a**, **b** Distribution of shoot-Na^+^ contents among 419 maize inbred lines under conditions with 100 mM NaCl (**a**) or 100 mM NaHCO_3_ (**b**). **c** Comparison of the shoot Na^+^ contents under NaCl and NaHCO_3_ conditions. The box shows the median, lower and upper quartiles, and dots denote outliers. Statistical significance was determined by a two-sided *t*-test (*n* = 419). **d** The relative change in shoot Na^+^ contents under NaHCO_3_ condition as compared with NaCl condition. The data were expressed as (*m* − *n*)/*n*. *m* and *n* referred to the shoot Na^+^ contents under NaHCO_3_ and NaCl condition respectively. **e**, **f** GWAS results of shoot Na^+^ contents under NaCl (**e**) and NaHCO_3_ (**f**) condition. A 2.5 Mb region (Chr2: 10.5–13.0 Mb) was displayed. Two SNPs (Chr2_12130275 and Chr2_12130134) that showed significantly association with shoot Na^+^ content under NaHCO_3_ condition were highlighted in red, and the gene underlies the association was designated as *ZmNSA1* (Na^*+*^ Content under Saline-Alkaline Condition). **g** The distribution of shoot Na^+^ contents. Statistical significance was determined by a two-sided *t*-test (*n* = 375 for genotype C; *n* = 23 for genotype T). The samples were grouped according to the haplotypes of SNP Chr2_12130275. Source data underlying Fig. 1d, g are provided as a Source Data file.

In this study, we aimed to identify factors regulating maize shoot Na^+^ homeostasis under saline-alkaline conditions. Given sodium hydrogen carbonate (NaHCO_3_) is one of the major basic salts in nature environments^30^, we used 100 mM NaHCO_3_ to mimic the saline-alkaline stress, and both the Na^+^ concentration (100 mM) and pH value (pH 8.8) were agronomic relevance^12,32^. Firstly, we compared the shoot Na^+^ contents in maize seedlings grown under NaHCO_3_ and neutral salt (NaCl) conditions. We grew 419 maize inbred lines under conditions with 100 mM NaHCO_3_ or 100 mM NaCl (pH 7.0) for two weeks, then measured the shoot Na^+^ contents (see Materials and methods), subsequently observed large variations of shoot Na^+^ contents ranging from 0.4 to 35 mg g^−1^ dry mass (Fig. 1a, b; Supplementary Data 1). The overall shoot Na^+^ contents of the plants grown under NaHCO_3_ condition were significantly greater than that grown under NaCl condition (*P* = 2.53 × 10^−66^; Fig. 1c), with 95% of the inbred lines conferred greater shoot Na^+^ contents under NaHCO_3_ condition than under NaCl condition (Fig. 1d). In addition, although different inbred lines showed large variations of shoot K^+^ contents (ranging from 22 to 83 mg g^−1^ dry mass) (Supplementary Fig. 1), the overall shoot K^+^ contents of the plants grown under NaHCO_3_ condition were comparable with that grown under NaCl condition (Supplementary Fig. 1c). Since the major feature distinguishing saline-alkaline stress from saline stress is the high pH stress, we suggest that high pH stress boosts maize shoot Na^+^ accumulation under high-Na^+^ conditions. Such a perspective is further supported by the observation that the increase of soil pH (from 7.0 to 10.0) caused up to 50% reduction of shoot biomass and up to 30% increase in shoot Na^+^ contents under condition with 100 mM NaCl (Supplementary Fig. 2).

### *ZmNSA1* confers natural variations of shoot Na^+^ contents

We next thought to identify the genetic variations underlying natural variations of maize shoot Na^+^ contents under NaHCO_3_ condition. GWAS analyses were performed using a mixed linear model (MLM; TASSEL 3.0) to identify the SNPs that were significantly associated with shoot Na^+^ content under either NaHCO_3_ or NaCl condition (see Materials and methods). Among the significant SNPs (−log_10_(*P*) > 5.0) (Supplementary Fig. 3), two SNPs next to each other (Chr2_12130275 and Chr2_12130134) were significantly associated with shoot Na^+^ contents under NaHCO_3_ condition but not under NaCl condition (Fig. 1e, f; Supplementary Fig. 3), with Chr2_12130275 showed greater association (−log_10_(*P*) = 5.5). We designated the gene underlies this significant association as *Zea may L. Na*^*+*^ *Content 1 under Saline-Alkaline Condition* (*ZmNSA1*), which potentially identifies an important mechanism regulating Na^+^ homeostasis under saline-alkaline condition. The leading SNP Chr2_12130275 was located in the 3′ untranslated region (3’UTR) of *GRMZM2G000397* (Supplementary Fig. 4), at which a thymine (T) and a cytosine (C) were associated with a lower and a greater shoot Na^+^ content respectively (Fig. 1g). *GRMZM2G000397* encodes a putative calcium-binding family protein, which contains a single EF-hand domain, but with no other domains of known function (Supplementary Fig. 5). The orthologues of ZmNSA1 were identified in most plant species (Supplementary Fig. 6), but their function remains unknown. The phylogenetic analysis indicated that ZmNSA1 and its othologues likely have evolutionary relationship with CML family protein (Supplementary Fig. 7), however, they haven’t been classified as CML family proteins in previous analysis^33^. Give previous studies have shown that saline-alkaline stress induces the increase of cytosolic Ca^2+^, which is perceived by Ca^2+^-binding proteins (e.g., the EF-hand containing proteins) then triggers downstream adaptive responses^6^, it is possible that the calcium-binding EF-hand protein encoded by *GRMZM2G000397* may perceives the saline-alkaline induced Ca^2+^ signal, then confers the regulation of Na^+^ homeostasis. Therefore, we suggest that *GRMZM2G000397* is a likely candidate of *ZmNSA1*.

**Fig. 2:**
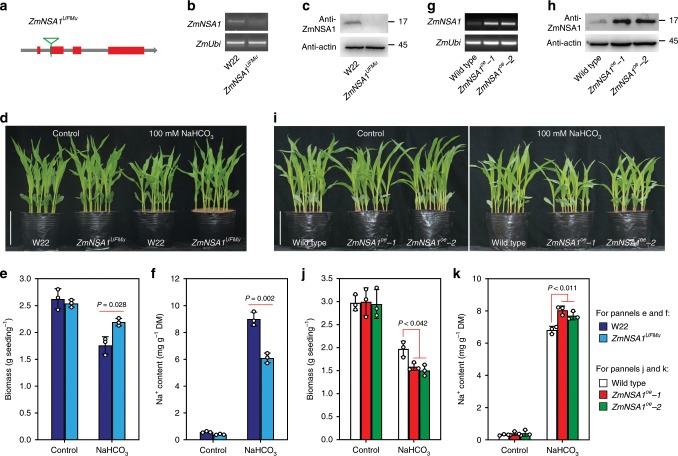
Lacking and overexpressing of *ZmNSA1* confer tolerance and hypersensitivity to saline-alkaline stress respectively. **a** Carton showed the site of the UniformMu insertion (highlighted by the green triangle) in *ZmNSA1*^*UFMu*^. **b** Semi-quantitative RT-PCR analysis of *ZmNSA1* transcript levels in *ZmNSA1*^*UFMu*^ and wild type (W22) plants. **c** Western blot analysis of ZmNSA1 protein levels in W22 and *ZmNSA1*^*UFMu*^. Similar results were seen in three independent experiments. **d**-**f** Appearances (**d**), biomasses (**e**), and shoot Na^+^ contents (**f**) of 2-weeks-old W22 and *ZmNSA1*^*UFMu*^ plants (growth conditions as indicated). **g**, **h** Transcript (**g**) and protein (**h**) levels of ZmNSA1 in *ZmNSA1*^*oe*^*-1*, *ZmNSA1*^*oe*^*-2* and wild type. **i**-**k** Appearances (**i**), biomasses (**j**) and shoot Na^+^ contents (**k**) of 2-weeks-old plants (genotypes and treatments as indicated). Bars in **d**, **i** equaled to 15 cm. Data in **e**, **f**, **j**, **k** were means ± s.d. of three independent experiments. Statistical significance was determined by a two-sided *t*-test. DM, dry mass. Analysis of actin provided a loading control in **c**, **h**. Source data underlying Figs. 2b, 2c, 2e–h, 2j, and 2k are provided as a Source Data file.

To determine if the candidate of *ZmNSA1* (*GRMZM2G000397*) is associated with shoot Na^+^ content and saline-alkaline tolerance in maize, we tried to generate *ZmNSA1* knockout mutant using previously described CRISPR-Cas9 technology^34,35^. Nevertheless, with four CRISPR-Cas9 targets and more than 80 independent transgenic plants (Supplementary Fig. 8), we failed to obtain *ZmNSA1* knockout line, which might be the consequence of low mutational efficiency^36^. Fortunately, we identified a mutant line (*ZmNSA1*^*UFMu*^), which conferred a UniformMu insertion in the second exon of *ZmNSA1* (Supplementary Fig. 9; Fig. 2a). *ZmNSA1* transcript and protein were hardly detected in *ZmNSA1*^*UFMu*^ mutant (Fig. 2b, c), indicating that *ZmNSA1*^*UFMu*^ conferred a function null allele of *ZmNSA1*. *ZmNSA1*^*UFMu*^ and wild type (W22) plants showed undetectable differences under control condition, but *ZmNSA1*^*UFMu*^ plants were significantly larger and conferred lower shoot Na^+^ content than that of W22 under NaHCO_3_ (100 mM) condition (Fig. 2d–f). Moreover, we generated two independent *ZmNSA1-*overexpressing lines (*ZmNSA1*^*oe*^*-1* and *ZmNSA1*^*oe*^*-2*), with both lines showed increased transcript and protein levels of ZmNSA1 (Fig. 2g, h). We observed that, while the growth of wild type and *ZmNSA1*-overexpressing plants were comparable under control condition, *ZmNSA1*^*oe*^*-1* and *ZmNSA1*^*oe*^*-2* plants were significantly smaller and conferred greater shoot Na^+^ contents than wild type under NaHCO_3_ condition (Fig. 2i–k). Taken together, these results indicated that ZmNSA1 is associated with shoot Na^+^ content and saline-alkaline (NaHCO_3_) tolerance, supporting the perspective that *GRMZM2G000397* is the candidate of *ZmNSA1*.

### InDel1032 reduces the translation efficiency of *ZmNSA1* mRNA

**Fig. 3:**
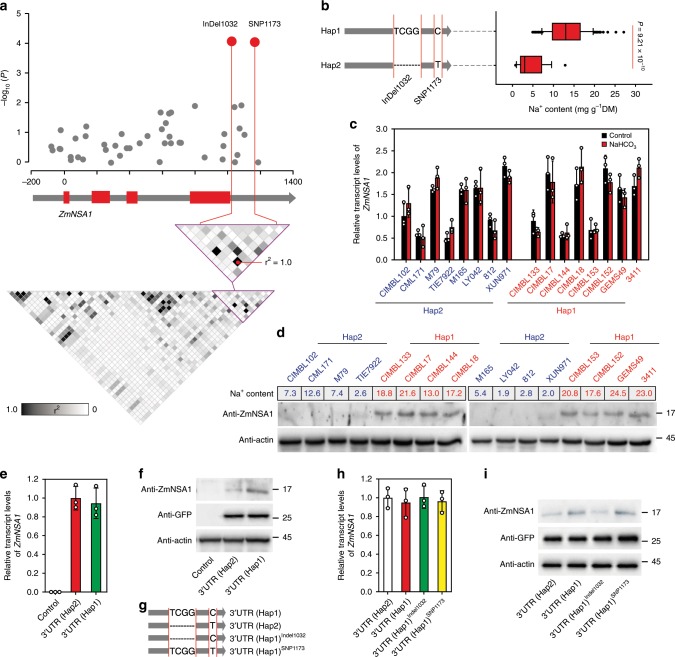
InDel1032 promotes shoot Na^+^ exclusion by decreasing the translation efficiency of *ZmNSA1* mRNA. **a** The association analysis of the genetic variations in *ZmNSA1* with shoot Na^+^ contents among 166 maize inbred lines. Upper panel showed the characterization of variations significantly associated with shoot Na^+^ contents. The red dots highlighted InDel1032 and SNP1173 that showed the highest association. Lower panel displayed the pattern of pairwise LD of the variations, and the red dots highlight the complete LD between InDel1032 and SNP1173. **b** Left panel showed the haplotypes of *ZmNSA1* grouped according to the significant variants. Right panel showed the distributions of shoot Na^+^ contents for each haplotype group. Statistical significance was determined by a two-sided *t*-test (*n* = 148 for Hap1; *n* = 18 for Hap2). **c**, **d** Comparison of the transcript (**c**) and protein (**d**) levels of ZmNSA1 between randomly selected Hap1 and Hap2 inbred lines under control and NaHCO_3_ conditions. **e**, **f** The transcript (**e**) and protein (**f**) levels of ZmNSA1 in *ZmNSA1*^*UFMu*^ protoplasts transformed with *pSUPER-ZmNSA1-3*′*UTR(Hap1)* and *pSUPER-ZmNSA1-3*′*UTR(Hap2)* (see Materials and methods). The non-transformed protoplasts provided a control. **g** Carton displayed the wild type and mutant 3′UTR forms. **h**, **i** The transcript (**h**) and protein (**i**) levels of *ZmNSA1* in *ZmNSA1*^*UFMu*^ protoplasts expressing *pSUPER-ZmNSA1* with indicated 3′UTRs. In **f**, **i**, co-transformation of *pSUPER-GFP* and subsequent protein blot assay using GFP antibody provided a control of transformation efficiency. Analysis of actin provided loading controls in **d**, **f**, **i**. Data in **c**, **e**, **h** were means ± s.d. of three independent experiments. Source data underlying Figs. 3c–f, 3h, and 3i are provided as a Source Data file.

In order to determine the molecular basis of the functional variation of *ZmNSA1*, we amplified and resequenced *ZmNSA1* from 166 maize inbred lines using two pairs of primers (ZmNSA1-G-F1/ZmNSA1-G-R1 and ZmNSA1-G-F2/ZmNSA1-G-R2) (Supplementary Data 2, 3), subsequently identified 50 SNPs and 3 InDels with minor allele frequency (MAF) above 5% (Supplementary Data 4). The association of these variations with shoot Na^+^ contents were analyzed using TASSEL (see the Materials and methods), and the results indicated that a SNP (SNP1173) and an InDel (InDel1032) showed the greatest association with shoot Na^+^ content (Fig. 3a). SNP1173 and InDel1032 were located in the 3′UTR of *ZmNSA1*, and they were in complete LD among the 166 inbred lines (Fig. 3a). Based on the haplotypes of SNP1173 and InDel1032, the 166 maize inbred lines were grouped into two haplotype groups (Hap1 and Hap2) (Fig. 3b; Supplementary Data 3). The Hap1 and Hap2 groups were composed of 148 and 18 inbred lines respectively, with Hap1 group conferred significantly higher shoot Na^+^ content than Hap2 group (Fig. 3b; *P* *=* 9.21 × 10^−10^). Therefore, the Hap1 and Hap2 *ZmNSA1* were designated as the high shoot Na^+^ (saline-alkaline-sensitive) and low shoot Na^+^ (saline-alkaline-tolerant) allele, respectively.

The functional variation of *ZmNSA1* could be due to transcriptional or posttranscriptional changes. We showed by qRT-PCR assay that Hap1 and Hap2 inbred lines showed comparable *ZmNSA1* transcript levels under both control and NaHCO_3_ conditions (Fig. 3c), suggesting unlikely that the functional variation of *ZmNSA1* was associated with the transcriptional change. In contrast, we found that Hap1 lines conferred significantly higher ZmNSA1 protein levels than Hap2 lines (Fig. 3d), suggesting likely that the functional variation of *ZmNSA1* was due to the change of protein abundance. Above observations revealed that SNP1173 and InDel1032 showed the greatest association with shoot Na^+^ content and were located in the 3′UTR of *ZmNSA1* (Fig. 3a). Given previous studies have indicated that messenger RNA with alternative 3′UTR isoforms can be translated with different efficiencies^37,38^, we then determined if SNP1173 and InDel1032 change the translation efficiency of *ZmNSA1* messenger RNA. We generated pSUPER-*ZmNSA1-3*′*UTR(Hap1)* and *pSUPER-ZmNSA1*-*3*′*UTR(Hap2)*, transformed them into *ZmNSA1*^*UFMu*^ protoplasts, and then compared the transcript and protein levels of ZmNSA1 (see Materials and methods). The results indicated that the protoplasts transformed with *pSUPER-ZmNSA1-3*′*UTR(Hap1)* and *pSUPER-ZmNSA1*-*3*′*UTR(Hap2)* conferred comparable *ZmNSA1* transcript levels, but the former produced significantly more ZmNSA1 protein than the later (Fig. 3e, f), suggesting that the 3′UTR(Hap1) confers greater translation efficiency than 3′UTR(Hap2). Moreover, two mutant 3′UTR(Hap1) isoforms resembling 3′UTR(Hap2) were generated, with 3′UTR(Hap1)^InDel1032^ conferred an 4-bp (TCGG) deletion and 3′UTR(Hap1)^SNP1173^ conferred a C to T substitution (Fig. 3g). We found that, while *ZmNSA1*^*UFMu*^ protoplasts transformed with *pSUPER-ZmNSA1-3*′*UTR(Hap1)*, *pSUPER-ZmNSA1*-*3*′*UTR(Hap2)*, *pSUPER-ZmNSA1-3*′*UTR(Hap1)*^*InDel1032*^ and *pSUPER-ZmNSA1*-*3*′*UTR(Hap1)*^*SNP1173*^ showed comparable transcript levels of *ZmNSA1* (Fig. 3h), the abundance of ZmNSA1 proteins in the *pSUPER-ZmNSA1-3*′*UTR(Hap1)*^*InDel1032*^-transformed protoplasts was significantly lower than *pSUPER-ZmNSA1*-*3*′*UTR(Hap1)*-transformed protoplasts, and was comparable with *pSUPER-ZmNSA1*-*3*′*UTR(Hap2)*-transformed protoplasts (Fig. 3i). Taken together, we suggest that the 4-bp (TCGG) deletion in the 3′UTR of Hap2 *ZmNSA1* reduces the translation efficiency of *ZmNSA1* mRNA, thus promoting shoot Na^+^ exclusion under saline-alkaline condition.

### Lacking of ZmNSA1 promotes root Na^+^ efflux

**Fig. 4:**
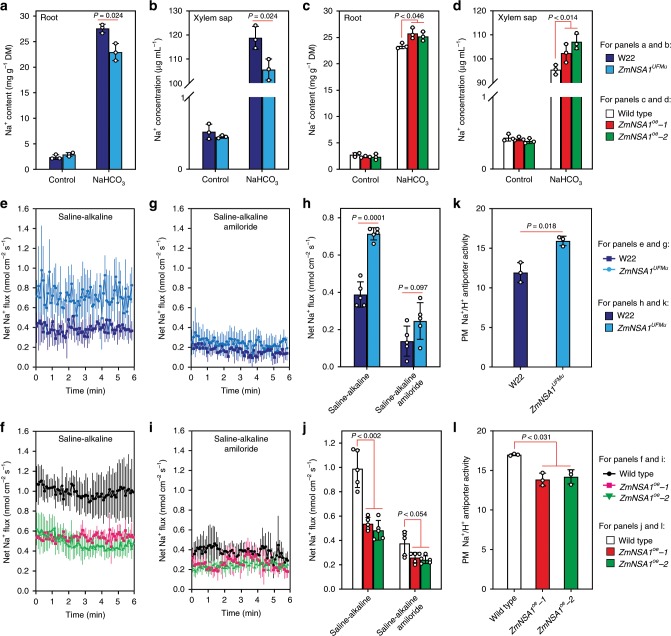
ZmNSA1 regulates root Na^+^ efflux under saline-alkaline conditions. **a**–**d** Na^+^ contents in the roots (**a**, **c**) and xylem sap (**b**, **d**) of *ZmNSA1*^*UFMu*^, *ZmNSA1*-overexpressing plants and their wild type controls (treatments as indicated). **e**–**j** Na^+^ flux at the root meristem zone of *ZmNSA1*^*UFMu*^, *ZmNSA1*-overexpressing plants and their wild type controls. Five-day-old plants were treated with 100 mM NaCl (pH 8.0) for 24-h, incubate in recording buffer (**e**, **f**) or recording buffer with 50 μM amiloride (**g**, **i**) for 30 mins, then the Na^+^ flux were measured using Non-invasive Micro-test Technology (NMT) (see Materials and methods). **k**, **l** The activity of Na^+^/H^+^ antiporter in the plasma membrane vesicles isolated from the roots of NaHCO_3_ treated plants (genotypes as indicated). Data in **a**–**d**, **k**, **l** were means ± s.d. of three independent experiments. Data in **e**–**j** were means ± s.d. *n* = 5. Statistical significances were determined by a two-sided *t*-test. Source data are provided as a Source Data file.

Above result has shown that lacking of ZmNSA1 promotes shoot Na^+^ exclusion under saline-alkaline condition (Fig. 2f), which could be ascribed to a decreased root Na^+^ uptake, or an increased root Na^+^ efflux, or a decreased root-to-shoot Na^+^ delivery. We then compared the Na^+^ contents in the root and xylem sap of *ZmNSA1*^*UFMu*^ and W22 (wild type) under NaHCO_3_ (100 mM) condition, and observed that *ZmNSA1*^*UFMu*^ conferred significantly lower root and xylem sap Na^+^ contents than W22 (Fig. 4a, b), suggesting likely that lacking of *ZmNSA1* decreases root Na^+^ content, thereby reducing xylem sap Na^+^ content and root-to-shoot Na^+^ delivery. In agree with this perspective, we observed that *ZmNSA1*-overexpressing plants conferred higher root and xylem sap Na^+^ contents than wild type under saline-alkaline condition (Fig. 4c, d).

We further determined how ZmNSA1 regulates root Na^+^ content, i.e. by decreasing uptake or by increasing efflux. Firstly, we used Non-invasive Micro-test Technology (NMT) to measure the Na^+^ flux at the root meristem zone of five-days-old seedlings that have been treated with 100 mM NaCl (pH 8.0) for 24 h, and observed that the roots of *ZmNSA1*^*UFMu*^ and *ZmNSA1*-overexpressing plants showed significantly greater and lower root Na^+^ efflux than that of the wild type controls respectively (Fig. 4e, f). Secondly, we measured the root Na^+^ contents of the plants that have been treated with 100 mM NaCl (pH 8.0) for short time (10 min), which to some extent reflects the rate of short-term Na^+^ uptake. The results indicated that the root Na^+^ contents in *ZmNSA1*^*UFMu*^ and *ZmNSA1*-overexpressing plants were comparable with that of their wild type controls (Supplementary Fig. 10). Taken together, these observations indicated that ZmNSA1 involves in the regulation of root Na^+^ efflux, but is unlikely associated with the regulation of Na^+^ uptake.

Previous studies have demonstrated that the root Na^+^ efflux is substantially mediated by SOS1 family Na^+^/H^+^ antiporters^7^, we then thought to determine if ZmNSA1 regulates root Na^+^ efflux by a Na^+^/H^+^ antiporter dependent mechanism. Amiloride is an inhibitor of Na^+^/H^+^ antiporter^39^. We found that, while *ZmNSA1*^*UFMu*^ conffered increased root Na^+^ efflux and *ZmNSA1*-overexpressing plants conferred decreased root Na^+^ efflux (Fig. 4e, f), the application of amiloride reduced root Na^+^ efflux of all tested genotypes, but with different degrees of reduction (Fig. 4e–j). As a result, amiloride application substantially reduced the differences of root Na^+^ efflux between *ZmNSA1*^*UFMu*^ and W22, and between *ZmNSA1*-overexpressing plants and wild type (Fig. 4e–j), suggesting that *ZmNSA1*-mediated regulation of root Na^+^ efflux is dependent upon SOS1 Na^+^/H^+^ antiporter. Such a perspective was supported by further obeservations, which showed that the plasma membrane vesicles isolated from the roots of NaHCO_3_ treated *ZmNSA1*^*UFMu*^ plants showed greater Na^+^/H^+^ antiporter activity than W22 (Fig. 4k), and the plasma membrane vesicles isolated from *ZmNSA1*-overexpressing plants conferred lower Na^+^/H^+^ antiporter activity than wild type (Fig. 4l). Taken together, we suggest that ZmNSA1 mediates the regulation of root Na^+^ efflux, with lacking of *ZmNSA1* increases SOS1 Na^+^/H^+^ antiporter-mediated root Na^+^ efflux, thereby promoting shoot Na^+^ homeostasis and saline-alkaline tolerance.

### Ca^2+^ binds to ZmNSA1 and triggers its degradation

**Fig. 5:**
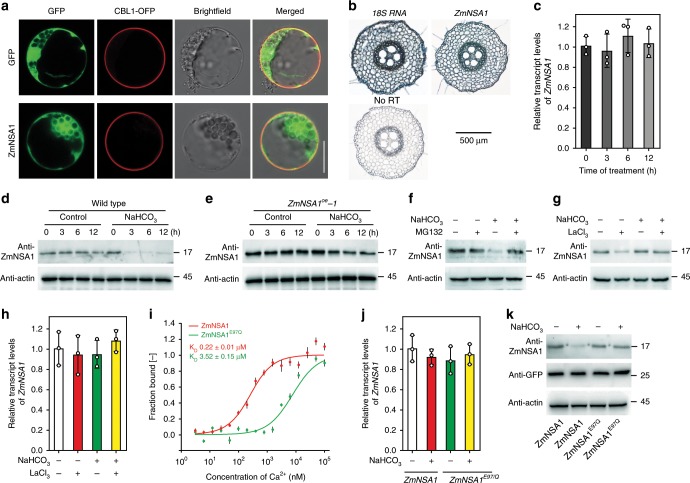
Ca^2+^ triggers the degradation of ZmNSA1 protein under saline-alkaline condition. **a** Subcellular localization of ZmNSA1-GFP in maize mesophyll protoplasts. CBL1-OFP is a marker for plasma membrane localization. Bars = 100 μm. **b** In situ RT-PCR analysis of the cell type specificity of *ZmNSA1* expression. The dark blue signal indicated the presence of *ZmNSA1* transcripts. The *18S rRNA* (*18S*) and no reverse transcription (no RT) provided positive and negative controls, respectively. **c** The effect of NaHCO_3_ treatment on the *ZmNSA1* transcript levels in the roots. **d**, **e** The effect of NaHCO_3_ treatment on ZmNSA1 protein levels in the root tissue of wild type (**d**) and *ZmNSA1*^*oe*^*-1* plants (**e**). In **c**–**e**, 2-weeks-old seedlings were subjected to the indicated treatments, and then the root tissues were collected at the indicated time points. **f**–**h** The influences of MG132 (**f**) and LaCl_3_ (**g**, **h**) treatments on ZmNSA1 protein (**f**, **g**) and transcript (**h**) levels. **i** MST assays of the Ca^2+^-binding affinity of ZmNSA1 and ZmNSA1^E97Q^ proteins. **j**, **k** The transcript (**j**) and protein (**k**) levels of ZmNSA1 in *ZmNSA1*^*UFMu*^ protoplasts transformed with *pSUPER-ZmNSA1*^*WT*^ and *pSUPER-ZmNSA1*^*E97Q*^ (treatments as indicated). Data in **c** and **h**–**j** were means ± s.d. of three independent experiments. Analysis of actin provided loading controls in **d**–**g**, **k**. Source data underlying Fig. 5c–k are provided as a Source Data file.

We next investigated the mechanisms by which ZmNSA1 responds to saline-alkaline stress then regulates SOS1 Na^+^/H^+^ antiporter-mediated root Na^+^ efflux. Firstly, we determined the subcellular localization of ZmNSA1, and found that ZmNSA1-GFP fusion proteins were predominantly detected in the cytosol of maize protoplast cells (Fig. 5a), but were hardly detected in nucleus (Supplementary Fig. 11). Secondly, the in situ RT-PCR assays showed that the transcripts of *ZmNSA1* were detected in all root cell types (Fig. 5b), and the saline-alkaline (100 mM NaHCO_3_) treatment for 3–12 h had insignificant effect on *ZmNSA1* transcription in the root tissues (Fig. 5c). Thirdly, the protein blot assays with an anti-ZmNSA1 antibody revealed that 100 mM NaHCO_3_ treatment for 3–12 h dramatically reduced the abundance of ZmNSA1 protein in the root tissues of wild type and *ZmNSA1*-overexpressing plants (Fig. 5d, e). Finally, we showed that the application of MG132 substantially inhibited the NaHCO_3_-induced degradation of ZmNSA1 (Fig. 5f). Taken together, these results indicated that NaHCO_3_ treatment triggers the degradation of ZmNSA1 protein via the 26S proteasome pathway, but has negligible effect on the transcript levels of *ZmNSA1*.

Previous studies have shown that Ca^2+^ is an important messenger mediating plant responses to environmental stresses^40,41^, and saline-alkaline treatment increases Ca^2+^ concentration in cytosol^6^. We then tested if the saline-alkaline induced degradation of ZmNSA1 is dependent upon the increase of cytosolic Ca^2+^. LaCl_3_ and verapamil are Ca^2+^-channel blocker^42,43^, which can block saline-alkaline stress induced increase of cytosolic calcium^6^. We found that the treatments with either 5 mM LaCl_3_ or 100 μM verapamil substantially inhibited the NaHCO_3_-induced degradation of ZmNSA1 (Fig. 5g; Supplementary Fig. 12a), but had undetectable effect on *ZmNSA1* expression and root cell variability (Fig. 5h; Supplementary Figs. 12b, 13), suggesting that saline-alkaline stress induced degradation of ZmNSA1 is dependent upon the increase of cytosolic Ca^2+^ concentration. Moreover, while LaCl_3_ treatment inhibited saline-alkaline treatment induced degradation of ZmNSA1 (Fig. 5g), the treatment reduced root Na^+^ efflux (Supplementary Fig. 14), which is consistent with above observations that *ZmNSA1*-overexpressing plants conferred decreased root Na^+^ efflux and SOS1 activity (Fig. 4f, l).

*ZmNSA1* encoded a putative calcium-binding EF-hand protein with a single EF-hand domain (Supplementary Fig. 5). Next, we investigated the Ca^2+^-binding profiles of ZmNSA1. The microscale thermophoresis (MST) assay indicated that ZmNSA1 binds Ca^2+^ directly (Fig. 5i), and a single amino acid change (ZmNSA1^E97Q^) in the conserved EF-hand domain resulted in a magnitude decrease of the Ca^2+^-binding activity (Fig. 5i), indicating that ZmNSA1 binds Ca^2+^ via the EF-hand domain. Notably, while previous studies have shown that, in a typical plant cell, free cytoplasmic Ca^2+^ concentrations are in the range of 100–200 nM, and increase to 500–1000 nM following the onset of external stimulations (e.g., salt stress)^44^, we observed that the Ca^2+^-ZmNSA1 binding increase linearly as Ca^2+^ concentrations increase from 100 to 1,000 nM (Fig. 5i), indicating that the Ca^2+^-ZmNSA1 binding is physiologically relevant. We further tested if the binding of Ca^2+^ is essential for the NaHCO_3_-induced degradation of ZmNSA1. We transformed *pSUPER-ZmNSA1* and *pSUPER-ZmNSA1*^*E97Q*^ into *ZmNSA1*^*UFMu*^ protoplasts, then examined the transcript and protein levels of ZmNSA1. The results indicated that ZmNSA1^E97Q^ mutation had undetectable effect on the expression of *ZmNSA1* (Fig. 5j), but significantly reduced the NaHCO_3_-induced degradation of ZmNSA1 (Fig. 5k). Taken together, we conclude that saline-alkaline stress (NaHCO_3_) increases cytosolic Ca^2+^, which binds to the EF-hand domain of ZmNSA1 then triggers its degradation, thereby promoting root Na^+^ efflux and promoting saline-alkaline adaptation.

### ZmNSA1 negatively regulates the activity of PM-H^+^-ATPase

**Fig. 6:**
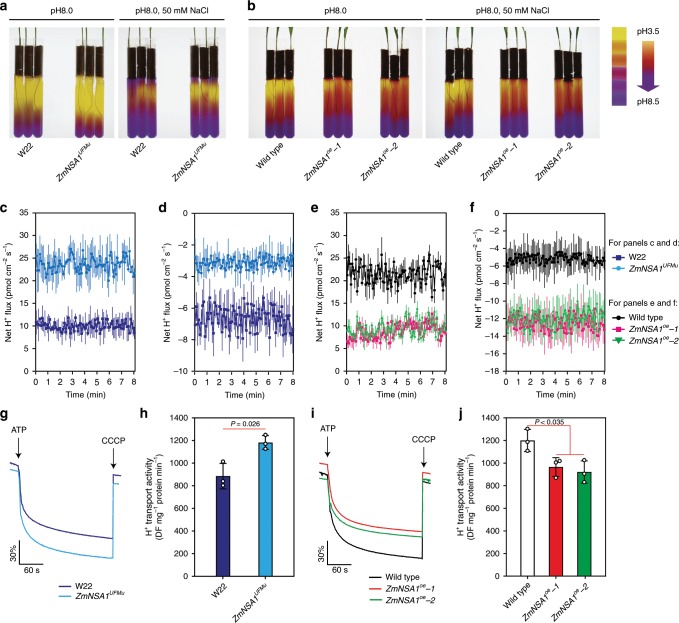
ZmNSA1 regulates PM-H^+^-ATPase-mediated H^+^ efflux. **a**, **b** Rhizosphere acidification assays of *ZmNSA1*^*UFMu*^ (**a**), *ZmNSA1*-overexpressing plants (**b**) and their wild type controls (W22 for *ZmNSA1*^*UFMu*^, and Wild type for *ZmNSA1*-overexpressing plants). Yellow color indicated the acidification of the medium. **c**–**f** NMT assays of H^+^ flux at root meristem zone of five-days-old seedlings that have been treated with alkaline (pH 8.0) (**c**, **e**) or saline-alkaline stress (100 mM NaCl, pH 8.0) (**d**, **f**) for 24 hours. The H^+^ flux were measured using NMT (see Materials and methods). Data were means ± s.d. *n* = 5. **g**–**j** The activity of PM-H^+^-ATPase in plasma membrane vesicles isolated from the roots of NaHCO_3_ treated plants (genotypes as indicated). The assays were performed as described in Materials and Methods. The data showed the timely varying curves of quinacrine fluorescent intensity (**g**, **i**) and the calculated activity of PM-H^+^-ATPase (**h**, **j**). Data were means ± s.d. of three independent experiments. Statistical significance was determined by a two-sided *t*-test. Source data underlying Fig. 6c–j are provided as a Source Data file.

Previous studies have shown that the PM-H^+^-ATPase-mediated root H^+^ efflux is a major determinant of the membrane potential^22,23^, which activates SOS1 Na^+^/H^+^ antiporters then promotes root Na^+^ efflux and saline-alkaline tolerance^18^. The above results have shown that *ZmNSA1* regulates SOS1 Na^+^/H^+^ antiporter-mediated root Na^+^ efflux (Fig. 4). We then determined if ZmNSA1 regulates root Na^+^ efflux by an H^+^ efflux dependent manner. Firstly, we grown *ZmNSA1*^*UFMu*^, *ZmNSA1*-overexpression plants and wild type plants under alkaline (pH 8.0) and saline-alkaline (50 mM NaCl, pH 8.0) mediums with the pH indicator bromocresol purple, and observed clear acidification of the mediums by all genotypes under both conditions. However, *ZmNSA1*^*UFMu*^ showed greater activity of medium acidification than W22, and *ZmNSA1-*overexpressing plants showed lower activity of medium acidification than wild type (Fig. 6a, b), suggesting likely that the function of ZmNSA1 is negatively associated with root H^+^ efflux. To confirm this perspective, we measured the net H^+^ flux at the meristem zone of 5-days-old plants that have been treated with alkaline stress (pH 8.0) or saline-alkaline stress (100 mM NaCl, pH 8.0) for 24-h (see Materials and methods), and found that *ZmNSA1*^*UFMu*^ conferred greater H^+^ efflux than W22 (Fig. 6c, d), and *ZmNSA1*-overexpressing plants conferred lower H^+^ efflux than wild type (Fig. 6e, f), confirming that ZmNSA1 negatively regulates root H^+^ efflux.

The root H^+^ efflux is substantially mediated by PM-H^+^-ATPase^45^. We then isolated plasma membrane vesicles from the roots of NaHCO_3_ treated plants, and then measured the activity of PM-H^+^-ATPase (Fig. 6g–j). The results indicated that *ZmNSA1*^*UFMu*^ conferred greater PM-H^+^-ATPase activities than W22 (Fig. 6g, h), and *ZmNSA1*-overexpressing plants conferred lower PM-H^+^-ATPase activities than wild type (Fig. 6i, j). These results support the notion that ZmNSA1 influences root Na^+^ efflux by regulating PM-H^+^-ATPase-mediated H^+^ efflux. In addition, while previous studies have suggested that the V-H^+^-ATPase and V-H^+^-PPase in tonoplast also affect Na^+^ homeostasis^46^, we observed that the tonoplast vesicles isolated from the roots of NaHCO_3_ treated *ZmNSA1*^*UFMu*^ and W22 plants showed comparable V-H^+^-ATPase and V-H^+^-PPase activities (Supplementary Fig. 15), indicating that ZmNSA1 has minimal effect on the activities of V-H^+^-ATPase and V-H^+^-PPase.

### ZmNSA1 mediates transcriptional upregulation of *MHAs*

**Fig. 7:**
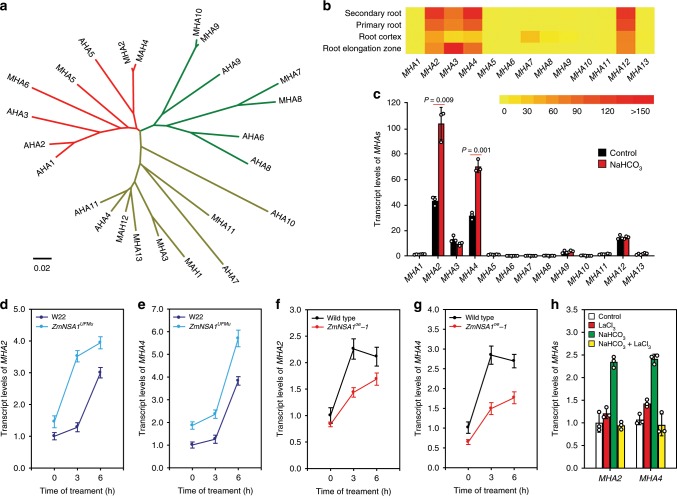
The degradation of ZmNSA1 confers transcriptional upregulation of *MHA2* and *MHA4*. **a** Phylogenetic tree of PM-H^+^-ATPase proteins from maize (MHA1-13) and *Arabidopsis* (AHA1-11). The phylogenetic tree was constructed using MEGA6^47^. **b** The transcript levels of 13 *MHAs* in the root tissues. The figure was generated from the data downloaded from https://www.maizegdb.org. **c** The effect of saline-alkaline stress (100 mM NaHCO_3_ for 6 hours) on the transcript levels of *MHAs* in the root tissues. Data were expressed as transcript levels relative to the *MHA1* transcription under control condition. **d**–**g** The transcript levels of *MHA2* and *MHA4* in the root tissues (genotypes and treatments as indicated). **h** The effect of NaHCO_3_ (100 mM) and LaCl_3_ (5 mM) treatments on the transcript levels of *MHA2* and *MHA4*. For **c**–**h**, 2-weeks-old plants were subjected to the indicate treatment, and then the root tissues were collected for analyzing the transcript levels of *MHAs*. Data in **c**–**g** were means ± s.d. of three independent experiments. Source data underlying Fig. 7c–h are provided as a Source Data file.

We next investigated the mechanism by which ZmNSA1 regulates the activity of PM-H^+^-ATPase. We showed that there were 13 *Maize PM-H*^*+*^*-ATPase*s (*MHA1*-*13*) (Fig. 7a; Supplementary Table 1), and the phylogenetic analysis using MEGA6^47^ showed that the PM-H^+^-ATPases from maize and *Arabidopsis* were grouped into three classes (Fig. 7a). The data from Maize Gene Expression Atlas showed that *MHA2*, *MHA3*, *MHA4*, and *MHA12* were predominantly detected in root tissues (Fig. 7b), which is in consistent with our qRT-PCR results (Fig. 7c). While previous studies have shown that the posttranscriptional activation of AHA2 increases root H^+^ efflux, and the Ca^2+^-binding 14-3-3 proteins directly binds and activates AHA2^6^, we didn’t observe the direct interaction between ZmNSA1 and MHA2 in yeast two-hybrid and BiFC assays (Supplementary Fig. 16). Intriguingly, we observed that NaHCO_3_ treatment significantly increased the transcript levels of *MHA2* and *MHA4* (the two most expressed *MHAs* in maize root tissues) (Fig. 7c), and such increases were enhanced in *ZmNSA1*^*UFMu*^ and attenuated in *ZmNSA1*-overexpressing plants (Fig. 7d–g), suggesting that ZmNSA1 mediates the transcriptional upregulation of *MHAs* under saline-alkaline condition. Moreover, while above studies have shown that LaCl_3_ treatment attenuated the NaHCO_3_-induced degradation of ZmNSA1 (Fig. 5g), the treatment also inhibited the NaHCO_3_-induced transcriptional upregualtion of *MHA2* and *MHA4* (Fig. 7h). Taken together, we conclude that, under saline-alkaline condition, Ca^2+^-triggered degradation of ZmNSA1 increases the transcript levels of *MHA2* and *MHA4*, then promotes root H^+^ efflux, thereby enhancing SOS1 Na^+^/H^+^ antiporter-mediated Na^+^ homeostasis and the adaptation to saline-alkaline environments.

## Discussion

Maize is a saline-alkaline sensitive crop, and global maize production is increasingly affected by the saline-alkalization of farmlands^29^. Therefore, there is an urgent need for understanding of maize saline-alkaline-tolerant mechanisms, understanding which can potentially be used to increase the saline-alkaline tolerance of maize. The sodium carbonates (NaHCO_3_ and Na_2_CO_3_) are the major basic salt existed in the saline-alkaline farmlands, which causes combined damages of high pH stress, Na^+^ toxicity and osmotic stress on maize^1^. Here, we have shown that natural maize inbred lines confer widely genetic variations of shoot Na^+^ exclusion under saline-alkaline (NaHCO_3_) condition (Fig. 1b), suggesting that the identification and application of the favorable variations minght provide a route for improving maize Na^+^ homeostasis and saline-alkaline tolerance. In addition, we have discovered that *ZmNSA1* underlies the natural variations of shoot Na^+^ contents under NaHCO_3_ condition (Fig. 1f). A naturally occurred 4-bp deletion decreases the transcription efficiency of *ZmNSA1* mRNA then promotes shoot Na^+^ exclusion (Fig. 3), accordingly, lacking of ZmNSA1 promotes shoot Na^+^ exclusion and saline-alkaline tolerance (Fig. 2d–f). Our identification of ZmNSA1 provides a gene target for improving maize saline-alkaline tolerance either by marker assisted selection or by CRISPR-Cas9 gene editing.

The major feature distinguishing saline-alkaline stress from saline stress is the high pH stress, which disturbs the H^+^ gradients across the plasma membrane. Under saline-alkaline condition, plants had to reinforce root-to-rhizosphere flux of H^+^, thus to establish the membrane potential^22,23^, which is important for the activation of H^+^-dependent sodium transporters (e.g., the SOS1 Na^+^/H^+^ antiporter)^6,18,45^. Previous studies have indicated that the PM-H^+^-ATPase is the major H^+^ pump responsible for root H^+^ efflux^45^, and that the posttranscriptional activation of PM-H^+^-ATPase (e.g., AHA2) confers Na^+^ homeostasis and saline-alkaline tolerance^6,45,48,49^. Here, we have shown that *MHA2* and *MHA4* were predominantly detected in root tissues and were significantly upregulated by saline-alkaline stress (Fig. 7b, c). These results together with previous studies suggest that the transcriptional and post-transcriptional activation of PM-H^+^-ATPase act together to ensure the establishment of the membrane potential under saline-alkaline conditions.

Existing knowledge have shown that Ca^2+^ is an important signaling molecule of saline-alkaline response^6^. Following the onset of saline-alkaline stress, the concentration of cytosolic-free Ca^2+^ increase, which then acts as a messenger to activate/inactivate the downstream signaling components^6^. The PM-H^+^-ATPase is one of the important downstream targets of Ca^2+^ signal, e.g., previous studies have shown that the Ca^2+^ binds to 14-3-3 proteins then mediates the posttranscriptional activation of AHA2^18^. We here show that the Ca^2+^-binding EF-hand family protein ZmNSA1 confers transcriptional regulation of maize PM-H^+^-ATPase (Fig. 7), which acts at the steps downstream of Ca^2+^ signal. ZmNSA1 negatively regulates the transcript levels of *MHAs*. Under saline-alkaline treatment, the concentration of cytosolic Ca^2+^ increase, then Ca^2+^ binds to the EF-hand domain of ZmNSA1 and triggers its degradation via 26S proteasome pathway (Fig. 5d–k), in turn promotes the transcription of *MHA2* and *MHA4* (Fig. 7). Given ZmNSA1 has no transcription activation domain and is barely detected in nucleus (Supplemental Fig. S5, 11), the ZmNSA1-mediated upregulation of *MHAs* transcript levels is likely to be an indirect response. SOS1 Na^+^/H^+^ antiporter is the major transporter responsible for root Na^+^ efflux^7^. We show that ZmNSA1 involves in the regulation of Na^+^/H^+^ antiporter-mediated root Na^+^ efflux, with lacking of ZmNSA1 increases the activity of Na^+^/H^+^ antiporter (Fig. 4). As previous studies have demonstrated that PM-H^+^-ATPase-mediated root H^+^ efflux is essential for the activation of SOS1 Na^+^/H^+^ antiporter^18^, we suggest that ZmNSA1-mediated regulation of Na^+^/H^+^ antiporter activity is ascribed to its regulatory roles on the transcription of PM-H^+^-ATPases.

**Fig. 8:**
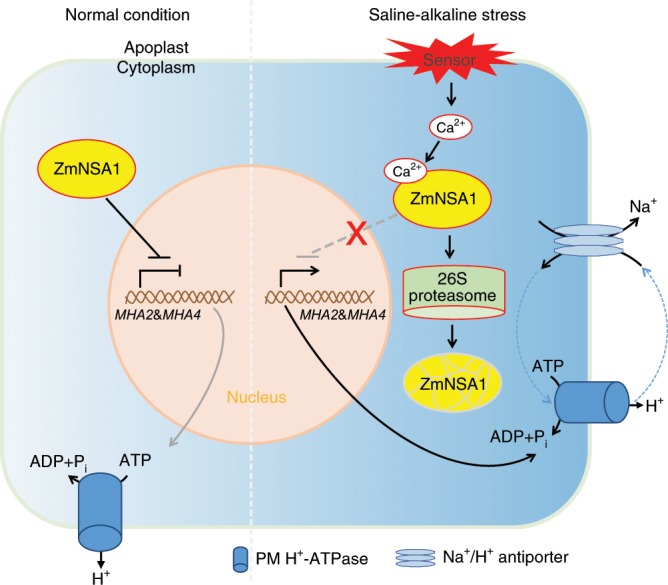
Working model of ZmNSA1-mediated regulation of Na^+^ homeostasis. Under normal growth condition, ZmNSA1 negatively regulates the transcript levels of *MHAs*. Under saline-alkaline condition, Ca^2+^ binds ZmNSA1 and triggers its degradation via 26S proteasome, then increases the transcript levels of *MHAs*, thereby enhancing root H^+^ efflux and promoting Na^+^ efflux mediated by SOS1 Na^+^/H^+^ antipoter.

In conclusion, we have discovered *ZmNSA1*, an important QTL conferring natural variations of shoot Na^+^ contents under saline-alkaline (NaHCO_3_) condition, with which we have discovered a saline-alkaline tolerance mechanism (Fig. 8), i.e., under saline-alkaline treatment, the concentration of cytosolic Ca^2+^ increase, Ca^2+^ binds to ZmNSA1 and triggers its degradation via the 26S proteasome pathway, then increases the transcript levels of maize PM-H^+^-ATPases (*MHA2* & *MHA4*) and promotes root H^+^ efflux, thereby enhancing SOS1 Na^+^/H^+^ antiporter-mediated root Na^+^ efflux, ultimately promoting saline-alkaline tolerance. Our study provides a mechanistic understanding of Ca^2+^-mediated plant saline-alkaline tolerance and an important gene target for breeding saline-alkaline tolerant maize varieties.

## Methods

### Plant growth and treatments

The natural maize population used in this study was the same population used in previously study^50,51^. In order to measure the shoot Na^+^ and K^+^ contents of the 419 maize inbred lines, pots (diameter of 30 cm and height of 35 cm) filled with uniformly mixed substrate (www.pindstrup.com) were watered to soil saturation with 100 mM NaCl or 100 mM NaHCO_3_ solutions. Eight inbred lines (six plants for each) were planted in each pot, grown in a glasshouse for 2 weeks, and then the shoot tissues were collected for measuring Na^+^ and K^+^ contents.

### Measurement of Na^+^ and K^+^ contents

The samples were dried at 80 °C for 24 h, weighed, then incinerated in a muffle furnace at 300 °C for 3 h and 575 °C for 6 h. The ashes were dissolved in 10 mL 1% hydrochloric acid, appropriately diluted with 1% hydrochloric acid, and then Na^+^ and K^+^ contents were analyzed. In order to measure the Na^+^ and K^+^ contents in xylem sap, 2-weeks-old seedlings grown under control or 100 mM NaHCO_3_ conditions were de-topped with blade, the xylem sap exuding at the cut surface of the de-topped root system was collected by a micropipette every 15 min for 1 h. The contents of Na^+^ and K^+^ were analyzed using the 4100-MP AES device (Agilent, Santa Clara, CA, USA).

### Genome-wide association study

The genotype used in this study was generated by Maize SNP50 array (containing 56,110 SNPs), RNA-seq or by joint application of IBD (identity by descent) based projection and KNN (the k-nearest neighbor) algorithm, and in total 556,809 high quality SNPs (MAF ≥ 0.05) were selected to perform the GWAS analysis^52^. Association analysis for shoot Na^+^ content under NaCl and NaHCO_3_ conditions were conducted by the mixed linear model (MLM; TASSEL3.0)^53^. Both kinship (K) and population structure (Q) were taken into account to avoid spurious associations^53^. We used the *P* < 1.0 × 10^−5^ as the final significance cutoff in the association analysis.

### Characterization of *ZmNSA1*^*UFMu*^

We ordered the UniformMu line (*mu1089781*) from Maize Genetics COOP Stock Center. The mutant line has been suggested to confer a UniformMu insertion in the second extron of *ZmNSA1*. In order to confirm the UniformMu insertion, we designated three primers (UFMu-F, UFMu-R and UFMu-S-F) (Supplementary Fig. 9; Supplementary Data 2), with which we can obtain PCR products from W22 when using UFMu-F and UFMu-R as primers, and can obtain PCR products from *mu1089781* when using UFMu-S-F and UFMu-R as primers. Subsequently, we confirmed that *mu108978*1 conferred a UniformMu insertion in the second extron of *ZmNSA1* (Supplementary Fig. 9), and the mutant was designated as *ZmNSA1*^*UFMu*^.

### Generation of *ZmNSA1* overexpressing lines

We generated the transgenic lines overexpressing *ZmNSA1* at Center for Crop Functional Genomics and Molecular Breeding, China Agricultural University, Beijing. The coding sequence of *ZmNSA1* was cloned to PBCXUN vector, transformed into *Agrobacterium strain* EHA105 to infecting immature embryo of inbred line 32990700 (a maize inbred line with increased transformation efficiency), then the regenerate seedlings were obtained from the infected embryo. The homozygous overexpression lines were obtained by anti-herbicide selection of the self-pollinated T1, T2 and T3 generation plants.

### qRT-PCR assay

Total RNA was extracted using RNA prep pure plant kit (Tiangen, Beijing, China), then 1.5 μg RNA was used to synthesize first-strand cDNA using M5 Super qPCR RT kit with gDNA remover (Mei5 biotechnology, Beijing, China), and then qRT-PCR analysis was conducted using the 2 × Real time PCR Super mix (SYBRgreen) (Mei5 biotechnology, Beijing, China) on the ABI 7500 thermocycler (Applied Biosystems). *Ubi2* gene (UniProtKB/TrEMBL, Q42415) provided a control, and the 2^−ΔΔCt^ method was used to calculate the expression.

### *ZmNSA1* association mapping and linkage analysis

To identify the genetic variation responsible for the functional variation of ZmNSA1, we used two pairs of primers (ZmNSA1-G-F1/ZmNSA1-G-R1 and ZmNSA1-G-F2/ZmNSA1-G-R2) to amplify and sequence *ZmNSA1* from 166 inbred lines randomly selected from the population. A genomic region including the 5’ to 3’ UTR of *ZmNSA1* was analyzed. Multiple sequence alignments were performed using BIOEDIT (v.7.0.9.0; North Carolina State University, Raleigh, NC, USA), and the polymorphic sites (SNPs and InDels) (MAF ≥ 0.05) were extracted. The associations between the genetic variations and Na^+^ contents were analyzed using TASSEL 3.0, under the standard MLM.

### Protoplast-based assay

In order to determine the subcellular localization of ZmNSA1, we isolated maize mesophyll protoplasts^54^, generated *pSUPER-ZmNSA1-GFP* vector, and then co-transformed *pSUPER-ZmNSA1-GFP* with *pGPTVII-AtCBL1-OFP*^55^ or *35S-AtWrky40-mCherry*^56^ into the mesophyll protoplasts. AtCBL1 is a plasma membrane localized protein^55^, and AtWrky40 is located in nucleus^56^. Fluorescent signals were captured using a confocal laser scanning microscope (Carl Zeiss LSM710). The excitation was at 488 nm and the detection was between 515 and 530 nm for GFP, and the excitation was at 543 nm and the detection was over 570 nm for OFP and mCherry. In order to analyze the transcription and protein levels of ZmNSA1 in *ZmNSA1*^*UFMu*^ protoplasts, the indicated constructs were transformed into the protoplasts, cultured for 16 h, then the protoplasts were used for analyzing *ZmNSA1* transcript and protein levels (Fig. 3e–i), or used for saline-alkaline treatments and follow up analysis of *ZmNSA1* transcript and protein levels (Fig. 5j, k).

### Immunoblot assay

Total proteins were extracted using extraction buffer (10 mM Tris, pH 7.5, 2.5 mM EDTA, 2.5 mM EGTA, 150 mM NaCl, 10 mM dithiothreitol, 1 mM phenylmethylsulfonyl fluoride; 1% protease inhibitor cocktail; Roche), then the target proteins were analyzed by immunoblot analysis. The antibodies used in this study include anti-ZmNSA1 generated by Beijing Protein Innovation, anti β-Actin (CWBIO 01265/60205, 1/5000) and anti-GFP (ABclonal AE011/33345, 1/5000).

### Non-invasive micro-test technology

The net Na^+^ and H^+^ fluxes were measured using non-invasive micro-test technology (NMT) (Younger USA, LLC, MA, USA). In order to measure the net Na^+^ and H^+^ fluxes, five-day-old seedlings were treated with 100 mM NaCl solution (pH 8.0) for 24 h, then Na^+^ and H^+^ effluxes (Figs. 4e–j, g, h and 6d, f) were measured at primary root meristem zone (~500 μm from the root tip). In addition, the H^+^ fluxes at meristem zone of five-day-old seedlings treated with water (pH 8.0) for 24 h were analyzed (Fig. 6c, e). The NMT measurement procedures as follows: The backfilling solution (250 mM NaCl for Na^+^ measurement; 15 mM NaCl plus 40 mM KH_2_PO_4_ for H^+^ measurement) were filled into the pre-pulled and salinized micro sensor (⌀4.5 ± 0.5 μm, XY- CGQ -01) to a length of 1.0 cm, and then 50–60 μm LIXs (XY-SJ-Na for Na^+^ measurement; XY-SJ-H for H^+^ measurement) were filled into the tip of the micro sensor. The micro sensor was calibrated in the calibration liquid (0.1 mM CaCl_2_, 0.1 mM KCl, 0.3 mM MES, and 0.5 mM or 5.0 mM NaCl, pH 6.0 for Na^+^ measurement; 0.1 mM CaCl_2_, 0.1 mM KCl, 0.3 mM MES, pH5.5 or pH 6.5 for H^+^ measurement). The roots were incubated in the measuring solutions (0.1 mM CaCl_2_, 0.1 mM KCl, 0.3 mM MES, and 0.5 mM NaCl, pH 6.0 for Na^+^ measurement; 0.1 mM CaCl_2_, 0.1 mM KCl, and 0.3 mM MES, pH 7.0 for H^+^ measurement) for 10 min, then the Na^+^ or H^+^ net fluxes were measured and calculated using JCal V3.3 (Younger, USA)^18^.

### Assay of rhizosphere acidification

The kernels were sterilized in 75% ethanol for 5 min, washed with sterilized water for three times, then enclosed with seed coating agent for later use. The culture MS media contained 30 g L^−1^ sucrose, 8 g L^−1^ agar, and 0.004% bromocresol purple, with or without 50 mM NaCl (pH 8.0). In order to avoid contamination, 2-cm-thick isolating layer (autoclaved mixture of sand and vermiculite) was added on the top of culture medium. The pretreated kernels were embedded in the isolating layer with a depth of 1 cm, and then cultured in greenhouse. The acidification of the media was analyzed 8 days later.

### In situ PCR

In situ PCR was conducted as described below. The roots were sliced into 50 μm-thick sections using Microtome (Leica, Germany). Then the samples were transferred into 100 μl sterile water with RNase inhibitor (1 U per μl), added 8 U DNase and incubated at 25 °C for 20 min to eliminate the genomic DNA, and then stop the reaction by adding 15 mM EDTA and heating to 75 °C for 10 min. The cDNA were synthesis with gene-specific primers (ZmNSA1-cDNA for ZmNSA1 and Zm18S-cDNA for 18S ribosomal RNA; Supplementary Data 2), then PCR amplifications were conducted in a reaction system containing 1 × PCR buffer, 1.5 mM MgCl_2_, 200 μM dNTPs, 0.4 nM digoxigenin-11-dUTP (Roche), 0.5 μM primers and 2 U Taq DNA polymerase (Thermo Fisher, USA). Following the PCR amplification, the samples were washed twice for 5 min with PBS buffer, blocked for 30 min in 0.1% BSA, incubated for 1 h with 1.5 U alkaline phosphatase-conjugated anti-digoxigenin Fab (Roche), washed twice for 15 min with washing buffer (0.1 M Tris-HCl, 0.15 M NaCl, pH9.5), stained with BM Purple AP Substrate precipitating (Roche) for 40 min, then washed twice with water and photographed using a Olympus microscope (BX53). The primers and sequences were listed in Supplementary Data 2.

### MST assay

*ZmNSA1* and *ZmNSA1*^*E97Q*^ were cloning into pGEXT vector and then transformed into *Escherichia coli* (DE3) to express the GST-tagged recombinant proteins. The purified GST-ZmNSA1 or GST-ZmNSA1^E97Q^ proteins (10 μM) were then labeled by dye (NT-647-NHS) using a Pierce™ BCA Protein Assay Kit (Thermo Fischer Scientific). The labeled proteins were incubated with indicate concentrations of Ca^2+^ (ligands) for 10 min, then the samples were analyzed by Monolish NT.115 (NanoTemper Technologies) at 25 °C, 20% MST power and 20% LED power using hydrophobic capillaries (Polymicro Technologies). The displayed results in Fig. 5i were based on three biological replicates and analyzed by MO. Affinity Analysis software (V2.2.4)^57^.

### Measurement activities of H^+^-ATPase and Na^+^/H^+^ antiporter

The isolation of plasma membrane vesicles, and the measurement of the activities of H^+^-ATPase and Na^+^/H^+^ antiporter were as described below. Plasma membrane vesicles were isolated from the roots of 2-week-old plants that have been treated with 200 mM NaHCO_3_ for 2 days. Fifty μg of plasma membrane proteins were used to determine the activity of H^+^-ATPase and Na^+^/H^+^ antiporter. The quenching in the fluorescence of quinacrine (a pH-sensitive fluorescent probe) provides a measure of H^+^-ATPase activity^58^. Na^+^/H^+^ exchange activity was calculated based on Na^+^-induced dissipation^59^. The quinacrine fluorescence was measured using a Hitachi F-7500 imager.

### Measurement of the V-H^+^-ATPase and V-H^+^-PPase activities

Two-week-old plants were treated with 200 mM NaHCO_3_ for 2 days, and then collected the roots to isolated tonoplast vesicles using differential centrifugation (25/33/50% (w/w) sucrose gradients). The vesicles that sedimented at the interface between 25% and 33% sucrose were collected. Fifty micrograms of tonoplast proteins were used to measure the proton transporting activity of V-H^+^-ATPase and V-H^+^-PPase. The reaction substrate for V-H^+^-ATPase is ATP (3 mM), and for V-H^+^-PPase is PP_i_ (1 mM). The quenching of the fluorescence detected by Hitachi F-7500 imager was regarded as the H^+^-transport activity^60^.

### Reporting summary

Further information on research design is available in the Nature Research Reporting Summary linked to this article.

## Supplementary information

Supplementary Information

Peer Review

Reporting Summary

Description of Additional Supplementary Files

Supplementary Data 1

Supplementary Data 2

Supplementary Data 3

Supplementary Data 4

## Data Availability

Data supporting the findings of this work are available within the paper and its Supplementary Information files. A reporting summary for this Article is available as a Supplementary Information file. The datasets generated and analyzed during the current study are available from the corresponding author upon request. The source data underlying Figs. 1d, g, 2b, c, e–h, j, k, 3c–f, h, i, 4, 5c–k, 6c–j, and 7c–h, as well as Supplementary Figs. 2c–f, 9b, 10, 12, 14, 15, and 16c–f are provided as a Source Data file.

## Source data

Source Data
