# Supplementary material for: Natural variation of an EF-hand Ca2+-binding-protein coding gene confers saline-alkaline tolerance in maize
Source: Nat Commun. 2020 Jan 10;11:186. doi: 10.1038/s41467-019-14027-y (PMC6954252; doi:10.1038/s41467-019-14027-y)
Supplement: Supplementary file 1 — Supplementary Information [file 41467_2019_14027_MOESM1_ESM.docx]

**Natural variation of an EF-hand Ca^2+^-binding-protein coding gene confers saline-alkaline tolerance in maize**

Cao *et al.*

**
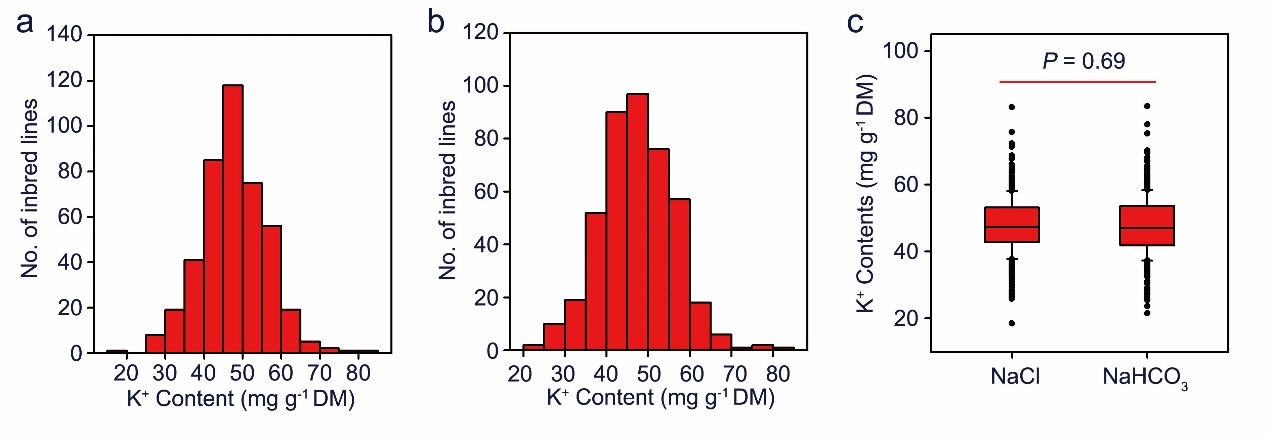
Supplementary Fig. 1. Distribution of shoot K^+^ contents.** **a**, **b** Distribution of shoot K^+^ contents among 419 maize inbred lines grown under conditions with 100 mM NaCl (**a**) or 100 mM NaHCO_3_ (**b**). **c** Comparison of the shoot K^+^ contents under NaCl and NaHCO_3_ conditions (*n* = 419). Statistical significance was determined by a two-side *t*-test.


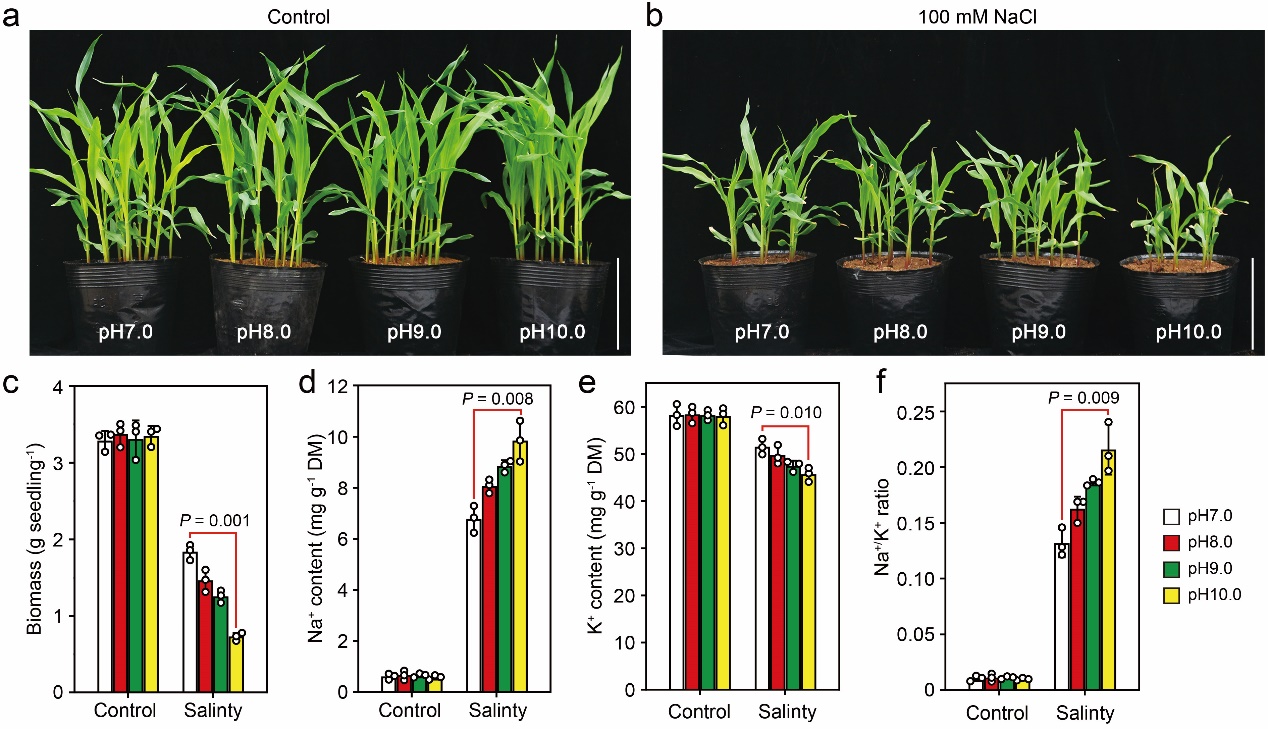


**Supplementary Fig. 2. High pH value worsens saline damage in maize. a**, **b** Appearances of two-weeks-old maize (B73) plants grown in soils saturated with water (Control) or 100 mM NaCl solution with indicated pH value. Bar = 15 cm. **c-f** Shoot biomasses (**c**), Na^+^ contents (**d**), K^+^ contents (**e**) and Na^+^/K^+^ ratios (**f**) of two-weeks-old maize seedlings grown in control and saline soils with indicated pH value. Data were **c**-**f** means ± s.d. of three independent experiments. Statistical significance was determined by a two-side *t*-test. Source data underlying Supplementary Figure 2c-f are provided as a Source Data file.

**
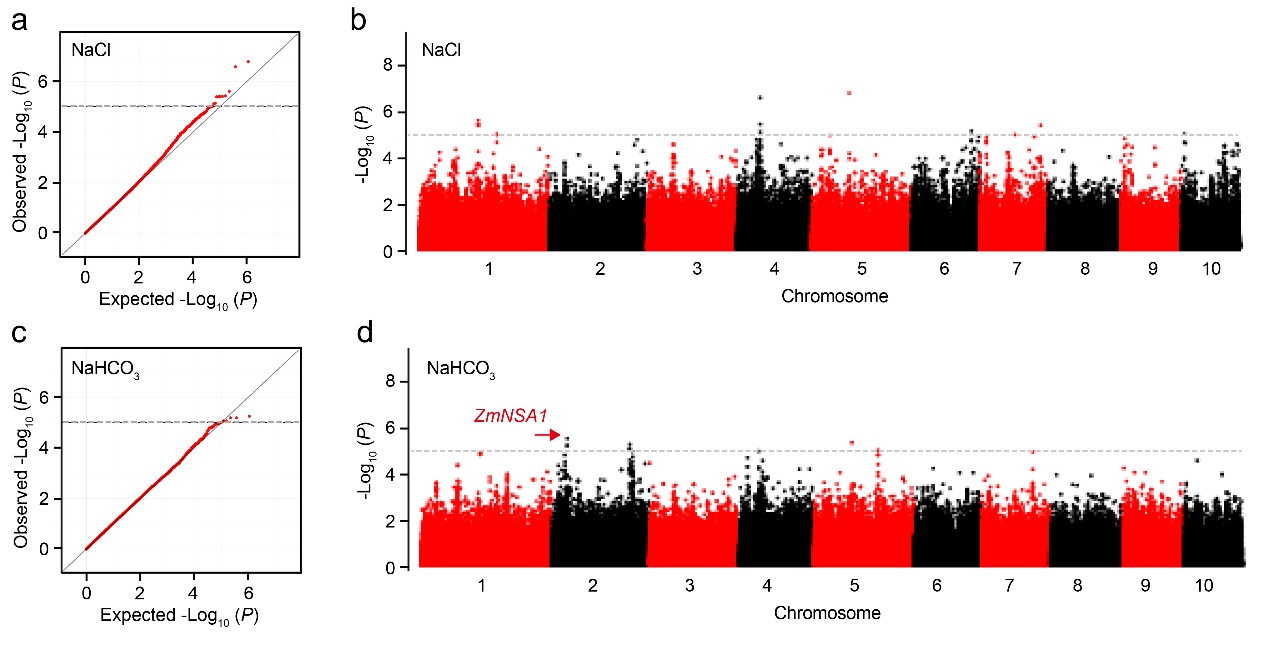
**

**Supplementary Fig. 3.** **GWAS for shoot Na^+^ content.** Quantile-quantile (QQ) plot (**a**, **c**) for the GWAS under a mixed linear model (MLM; TASSEL 3.0) and the Manhattan plot for the GWAS results (**b**, **d**). The dashed horizontal lines depict the significance threshold. The red arrow highlighted the location of *ZmNSA1* (*Na^+^ Content under Saline-Alkaline Condition*). The QQ plot of the expected and observed *P*-value suggests that there is no obvious inflation of the distribution of *P*-values.

**
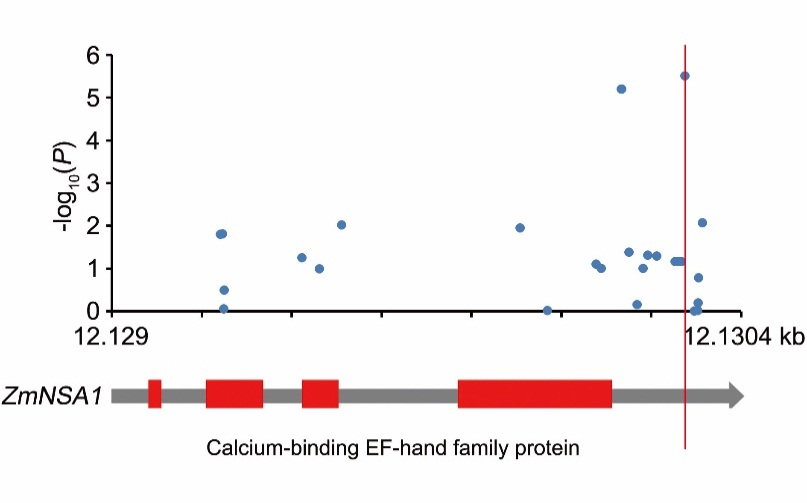
**

**Supplementary Fig. 4.** **The location of SNP Chr2_12130275.** The SNP Chr2_12130275 mapped to the 3’ untranslated region (3’UTR) of the *ZmNSA1* candidate (*GRMZM2G000397*).


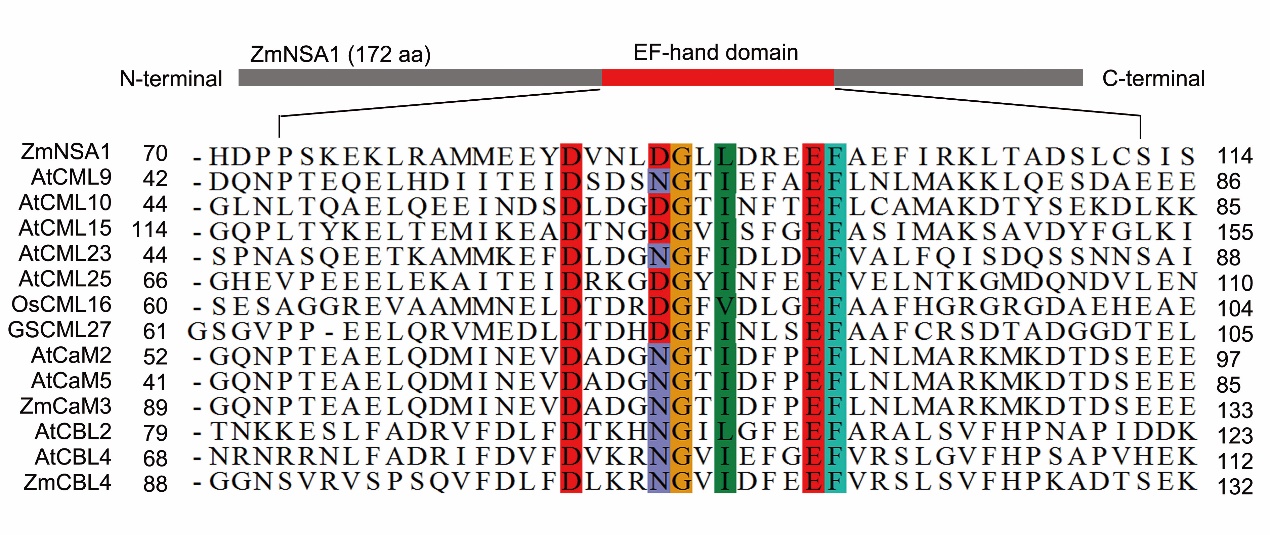
**Supplementary Fig. 5.** **Alignment of the EF-hand domain of ZmNSA1 and other selected proteins.** The amino acids highlighted in different colours showed the conserved amino acid within the EF-hand domain.


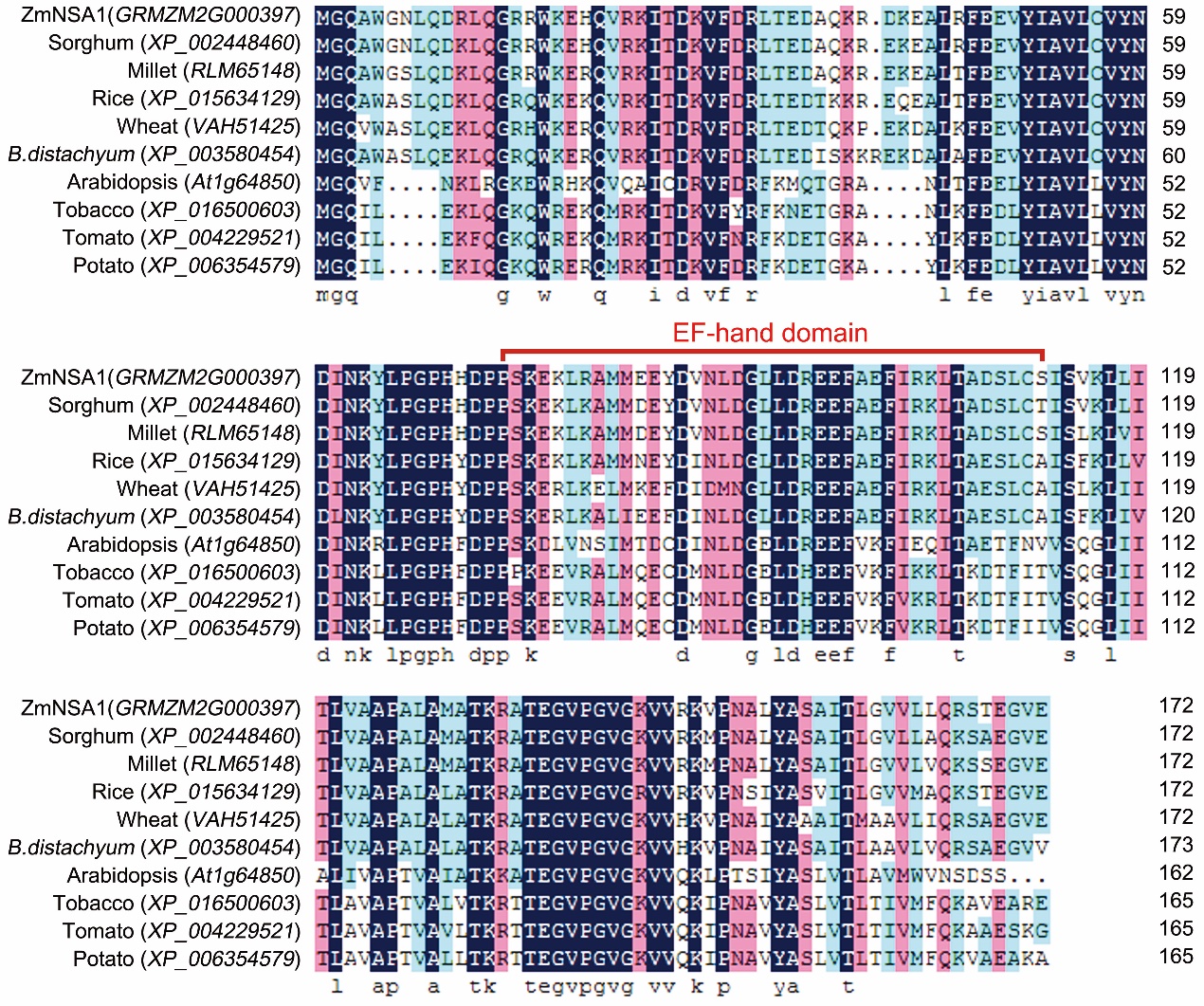


**Supplementary Fig. 6. Multiple-sequence alignment of ZmNSA1 orthologues.**


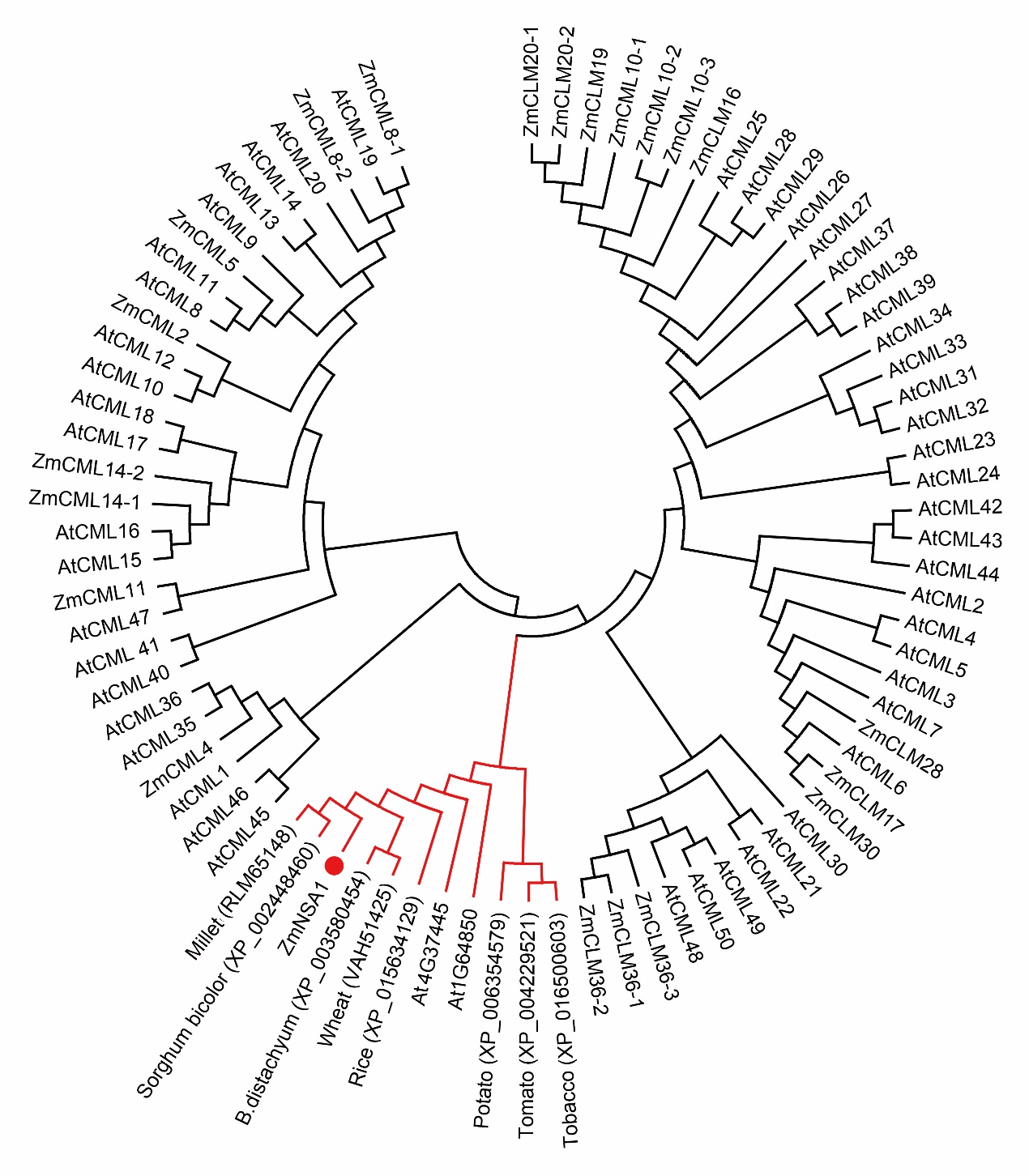


**Supplementary Fig. 7. Phylogenetic tree composed of ZmNSA1 othologues and selected CML proteins.** The CML proteins in maize and *Arabidopsis* were as described in previous study^1^. The red dot highlighted ZmNSA1, and the red lines highlighted the clade contains ZmNSA1 and its orthologues. The phylogenetic tree was constructed using MEGA6^2^.


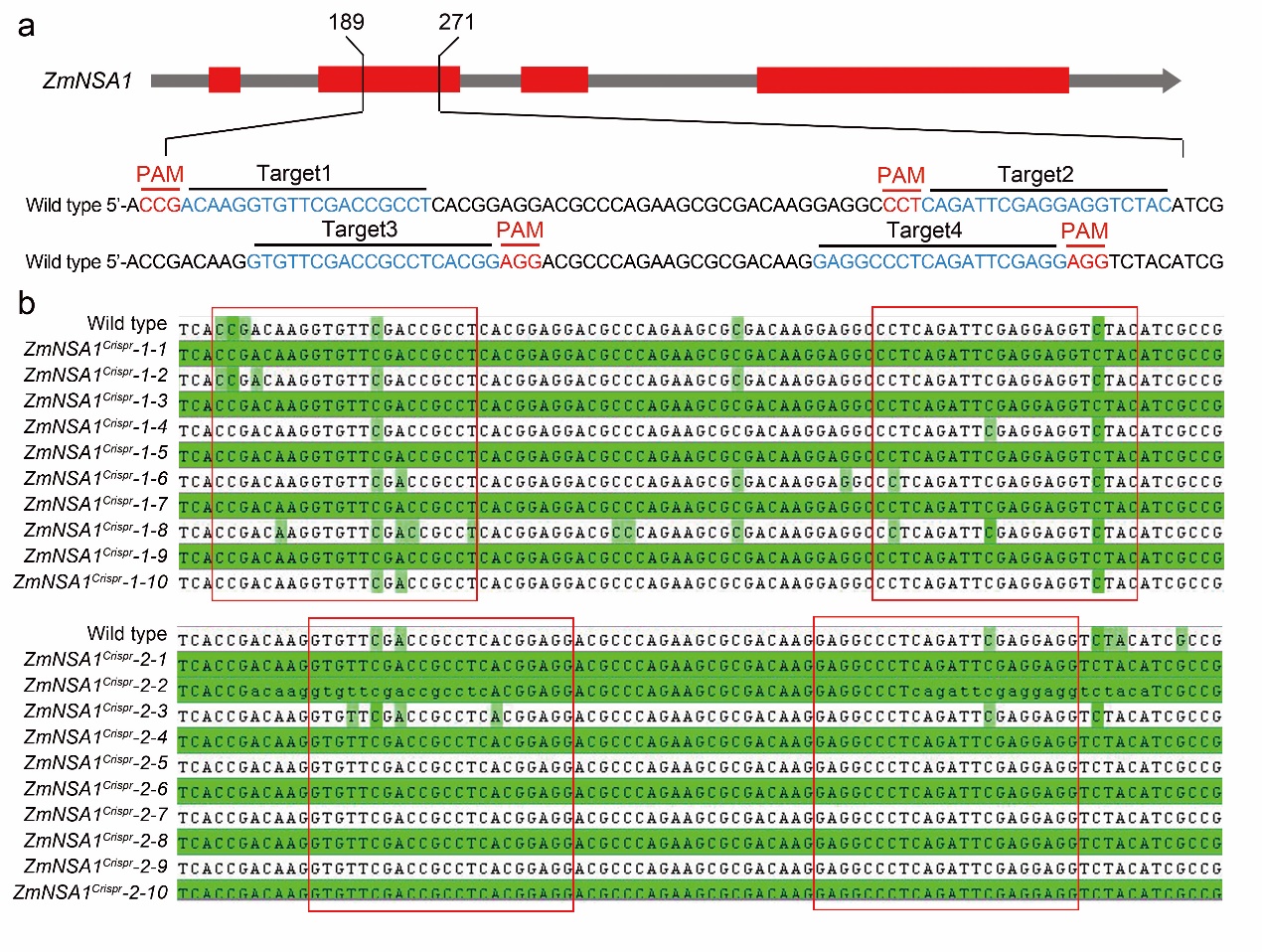


**Supplementary Fig. 8. The failure of generating *ZmNSA1* mutant via CRISPR-Cas9.** **a** Four gRNAs designed by CRISPR-P (http://crispr.hzau.edu.cn/CRISPR2/). **b** The sequencing results of the T0 generation *ZmNSA1^Crispr^* plants. Twenty randomly selected sequencing results were displayed. CRISPR/Cas9 targets were highlighted by red boxes.


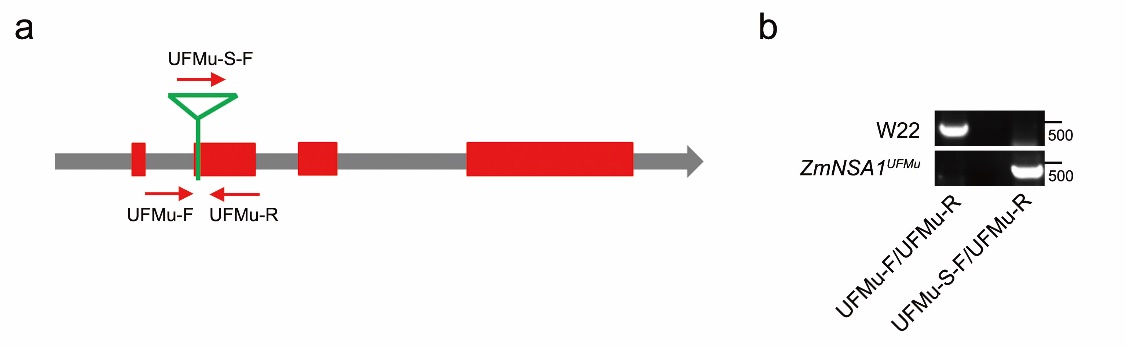


**Supplementary Fig. 9. The characterization of the homozygous *ZmNSA1^UFMu^* mutant.** **a** The site of the UniformMu insertion (highlighted by the green triangle) and the primers for genotyping. **b** The results of the PCR amplifications in wild type (W22) or homozygous *ZmNSA1^UFMu^* mutant. Similar results were seen in three independent experiments. Source data underlying Supplementary Figure 9b are provided as a Source Data file.


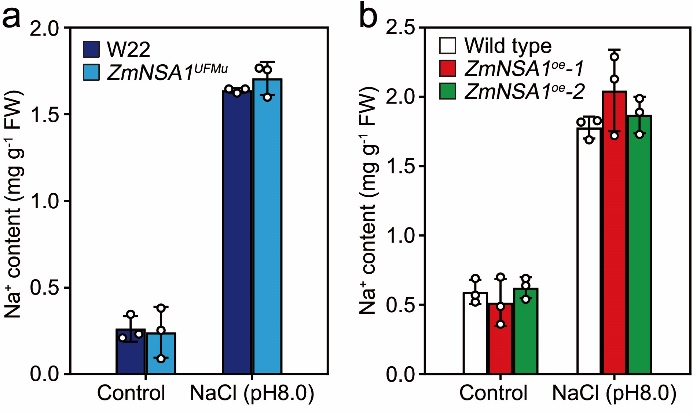


**Supplementary Fig. 10.** **Root Na^+^ contents.** Plants (genotypes as indicated) were grown for 5 days, treated with water (Control) or 100 mM NaCl (pH8.0) for 10 min, and then roots were collected for measuring Na^+^ contents. Data were mean ± s.d. of three independent experiments. Source data are provided as a Source Data file.


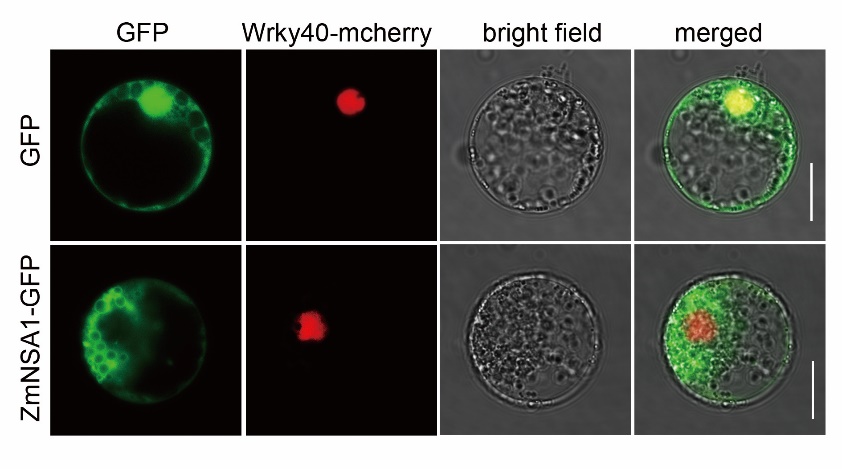


**Supplementary Fig. 11. Subcellular localization of ZmNSA1-GFP in maize protoplasts.** Maize mesophyll protoplasts were cotransformed with *pSUPER-ZmNSA1-GFP* and *35S-Wrky40-mCherry*, then the fluorescent signals were captured using a confocal laser scanning microscope (Carl Zeiss LSM710). Wrky40-mCherry was localized in nucleaus^3^. The results indicated that the colocalization of ZmNSA1-GFP and Wrky40-mCherry was barely detected. Similar results were seen in three independent experiments.


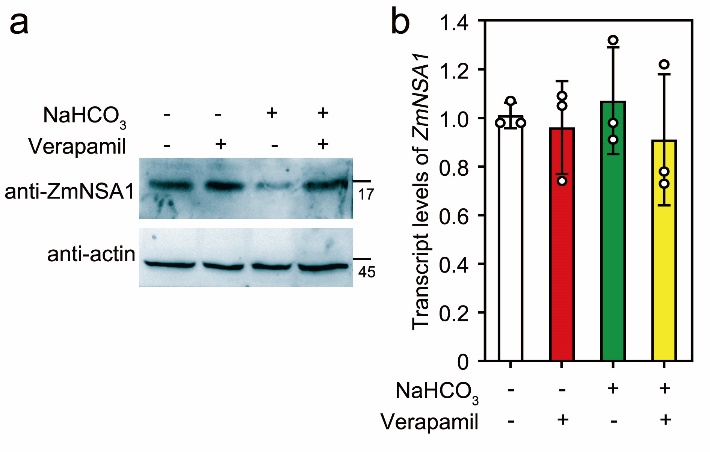


**Supplementary Fig. 12. The influences of verapamil on ZmNSA1 protein and transcript levels.** Two-weeks-old plants were subjected to indicated treatments for 3 hours (verapamil, 100 μM; NaHCO_3_, 100 mM), then the root tissues were collected to analyze the protein (**a**) and transcript (**b**) levels of *ZmNSA1*. Results similar to **a** were seen in three independent experiments. Data in **b** were means ± s.d. of three independent experiments. Source data are provided as a Source Data file.


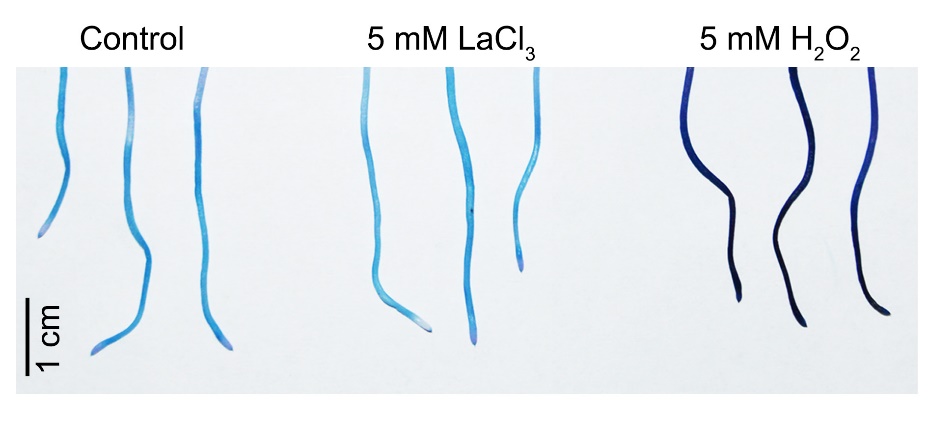


**Supplementary Fig. 13.** **Trypan blue staining of roots that have been treated with 5 mM LaCl_3_ for 3 hours.** The staining of the roots treated with water (Control) and 5 mM H_2_O_2_ provided negative and positive controls of cell death^4^. Similar results were seen in three independent experiments.


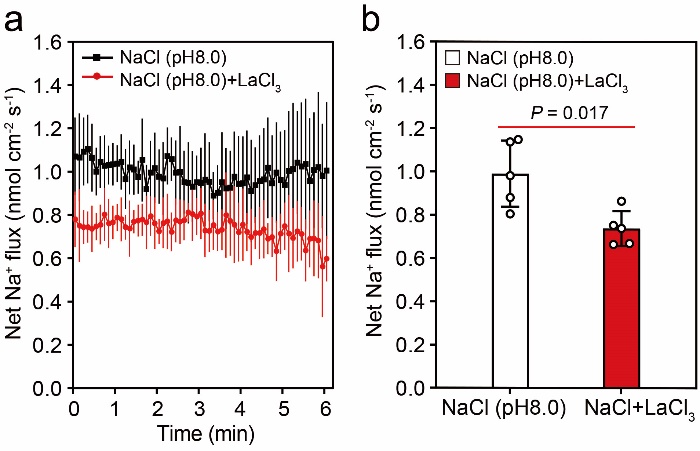


**Supplementary Fig. 14. Na^+^ flux at the root meristem zone of maize plants with indicated treatments.** Five-day-old plants were treated with 100 mM NaCl (pH8.0) for 24 h, then treated with LaCl_3_ for 30 minutes, and then the Na^+^ flux were measured using Non-invasive Micro-test Technology (NMT) (see Materials and methods). Data were means ± s.d. *n* = 5. Statistical significance was determined by a two-side *t*-test. Source data are provided as a Source Data file.


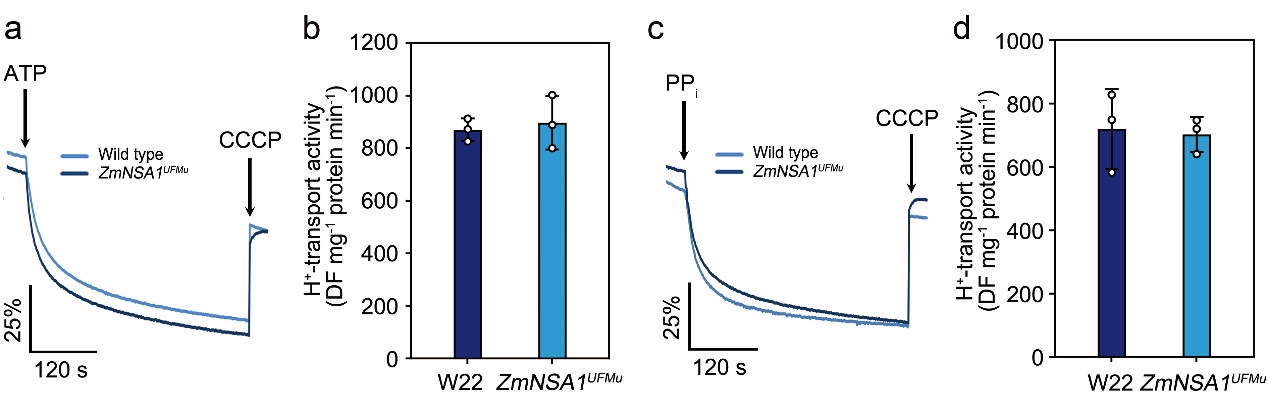


**Supplementary Fig. 15. The activities of V-H^+^-ATPase and V-H^+^-PPase in the tonoplast vesicles.** Tonoplast vesicles were isolated from roots of NaHCO_3_ treated plants (genotypes as indicated). Timely varying curves of quinacrine fluorescent intensity (**a**, **c**) and the calculated activity of V-H^+^-ATPase (**b**) and V-H^+^-PPase (**d**). Data in **b** and **d** were means ± s.d. of three independent experiments. Source data are provided as a Source Data file.


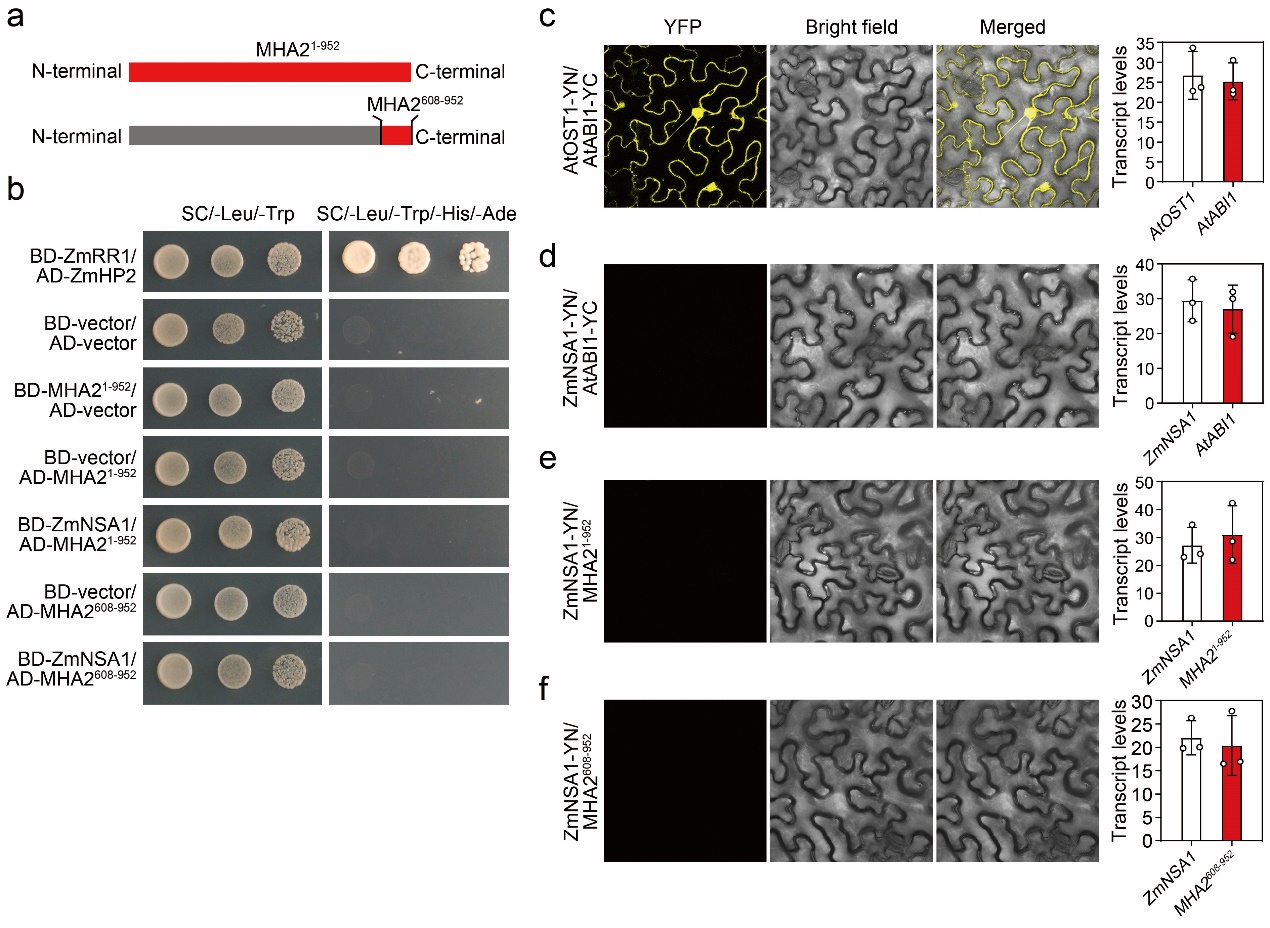


**Supplementary Fig. 16. Analysis of the interaction between ZmNSA1 and MHA2.** **a** Cartons showed the full length (MHA2^1-952^) or the C-terminal (MHA2^608-952^) of MHA2 used to determine the interaction between ZmNSA1 and MHA2. **b** The yeast two-hybrid assays didn’t detect the interaction between ZmNSA1 and MHA2. BD-ZmRR6/AD-ZmHP2 provided positive control^5^. **c-f** BiFC assays didn’t detect the interaction between ZmNSA1 and MHA2 in *N. benthamiana*. The YFP fluorescence was detected by confocal microscopy two days post-infiltration. Similar results were seen in three independent experiments. The transcript levels were expressed as transcript level of indicated genes relative to *ACTIN*^6^, and were means ± s.d. of three independent experiments. YN-AtOST1/YC-AtABI^7^ and YN-ZmNSA1/YC-AtBAI1 provided positive and negative controls respectively. Source data underlying Supplementary Figure 16c-f are provided as a Source Data file.

**Supplementary Table 1. List of the 13 - PM H^+^-ATPases encoded by maize genome.**

| **Gene name** | **Tracking ID** |
| --- | --- |
| MHA1 | GRMZM2G144821 |
| MHA2 | GRMZM2G019404 |
| MHA3 | GRMZM2G104325 |
| MHA4 | GRMZM2G006894 |
| MHA5 | GRMZM2G341058 |
| MHA6 | GRMZM2G008122 |
| MHA7 | GRMZM2G148374 |
| MHA8 | GRMZM2G455557 |
| MHA9 | AC209050.3_FG003 |
| MHA10 | GRMZM2G131309 |
| MHA11 | GRMZM2G172183 |
| MHA12 | GRMZM2G035520 |
| MHA13 | GRMZM2G068259 |

**Supplementary References**

1. Mohanta, T.K., Kumar, P. & Bae, H. Genomics and evolutionary aspect of calcium signaling event in calmodulin and calmodulin-like proteins in plants. *BMC Plant Biol* **17**, 38 (2017).
2. Tamura, K., Stecher, G., Peterson, D., Filipski, A. & Kumar, S. MEGA6: Molecular Evolutionary Genetics Analysis version 6.0. *Mol Biol Evol* **30**, 2725-2729 (2013).
3. Shang, Y. *et al.* The Mg-chelatase H subunit of *Arabidopsis* antagonizes a group of WRKY transcription repressors to relieve ABA-responsive genes of inhibition. *Plant Cell* **22**, 1909-1935 (2010).
4. Yao, N. *et al.* Novel evidence for apoptotic cell response and differential signals in chromatin condensation and DNA cleavage in victorin-treated oats. *Plant J* **28**, 13-26 (2001).
5. Asakura, Y. *et al.* Molecular characterization of His-Asp phosphorelay signaling factors in maize leaves: implications of the signal divergence by cytokinin-inducible response regulators in the cytosol and the nuclei. *Plant Mol Biol* **52**, 331-341 (2003).
6. Qi, T. *et al.* The Jasmonate-ZIM-domain proteins interact with the WD-Repeat/bHLH/MYB complexes to regulate Jasmonate-mediated anthocyanin accumulation and trichome initiation in *Arabidopsis thaliana*. *Plant Cell* **23**, 1795-1814 (2011).

7. Vlad, F. *et al.* Protein phosphatases 2C regulate the activation of the Snf1-related kinase OST1 by abscisic acid in *Arabidopsis*. *Plant Cell* **21**, 3170-3184 (2009).
