# Supplementary material for: Natural variation of an EF-hand Ca2+-binding-protein coding gene confers saline-alkaline tolerance in maize
Source: Nat Commun. 2020 Jan 10;11:186. doi: 10.1038/s41467-019-14027-y (PMC6954252; doi:10.1038/s41467-019-14027-y)
Supplement: Supplementary file 4 — Description of Additional Supplementary Files [file 41467_2019_14027_MOESM4_ESM.docx]

**Description of Additional Supplementary Files**

File name: Supplementary data 1
Description: Shoot Na^+^ and K^+^ contents of 419 maize inbred lines under NaCl or NaHCO_3_ condition.

File name: Supplementary data 2
Description: List of the primers used in this study.

File name: Supplementary data 3
Description: List of 166 maize inbred lines used in association mapping and linkage analysis.

File name: Supplementary data 4
Description: List of the natural genetic variations identified in *ZmNSA1*.
