# Supplementary material for: Natural variation of an EF-hand Ca2+-binding-protein coding gene confers saline-alkaline tolerance in maize
Source: Nat Commun. 2020 Jan 10;11:186. doi: 10.1038/s41467-019-14027-y (PMC6954252; doi:10.1038/s41467-019-14027-y)
Supplement: Supplementary file 2 — Peer Review [file 41467_2019_14027_MOESM2_ESM.pdf]

Reviewers' comments:

Reviewer #1 (Remarks to the Author):

Review for Manuscript NCOMMS-19-18400

In this submission Cao et al. describe their identification and functional characterization of the EF-hand containing Ca2+-binding protein ZmNCA1. The authors pursued an extensive GWAS-approach encompassing 419 maize inbred lines to identify genetic loci that specifically determine maize tolerance towards saline-alkaline stress. The association analysis identified a SNP in the 3' untranslated region of the NCA1 gene as candidate locus for affecting saline-alkaline tolerance. The authors, moreover, isolated a transposon-induced loss-of-function allele and generated overexpression lines. The phenotype analyses of these plants unambiguously established NCA1 as the locus important for saline-alkaline tolerance and provided evidence that the encoded protein modulates root Na+ uptake under stress conditions. Subsequently, the authors characterized mechanistic aspects of this function. To this end the authors used an elegant approach of transient protoplast transfection that revealed a role of the SNP in the 3' untranslated region in regulating translation efficiency of NCA1. Moreover, the authors provide experimental evidence that NCA1 is indeed a Ca2+ binding protein and that binding of Ca2+ appears to result in NCA1 degradation, a finding that is in line with the occurrence of salt stress induced Ca2+ signals in plants. Finally, the authors report that degradation of NCA1 confers transcriptional upregulation of specific plasma membrane ATPases. These findings allow the authors to propose a model (which unfortunately is not illustrated by a figure) in which saline-alkaline stress would trigger Ca2+ signals, leading to NCA1 degradation, allowing for enhanced root H+ efflux, counteracting this stress. The authors pursued all technically feasible approaches to illuminate further mechanistic details how NCA1 degradation impacts on ATPase activity (like direct protein interaction) and could finally provide a correlation between the transcriptional regulation of ATPases and NCA1 function.

Overall, the findings reported in this work are of greatest general relevance and clearly deserve reporting in a journal addressing a broad audience. The experiments are generally well executed and appropriate controls have been performed.

However, the current shape of the manuscript prevents publication. The manuscript appears to be hastily written and is full of mistakes with regards to grammar and writing. Several formulations are awkward to an extent that they interfere with the comprehensibility of the statements. The authors even confused "protoplasts" with "chloroplasts" and "nitrate" with "nitrite".

Further specific comments:

- The NCA1 protein is not well described. The molecular structure is only rudimentary explained. Moreover, it remains unclear if this protein belongs to a gene family and if related proteins exist in other plant species and if anything is known about the function of related proteins.
- The authors do not discuss if their determined Ca2+ binding properties of NCA1 would be in a range that could be physiologically relevant.
- The localization data presented in Fig. 5A may contain artefacts, since for example CBL1-OFP appears to decorate chloroplasts. This is likely a consequence of inappropriate microscopic analyses.
- The BiFC analyses (Supplemental Fig. 7) lack expression controls.
- The authors used a concentration of 5 mM LaCl3 to induce degradation of NCA1. Although reference concentrations for the application of La+ in maize are currently not available, such a concentration usually kills other plants. Therefore, the authors need to include controls that verify the viability of the La+ treated plants. On the other hand it would strengthen the conclusiveness of this assay, if the authors could provide any evidence that their La+ treatment indeed inhibits Ca2+ dependent processes of salt tolerance (like for example SOS1 activity).
- The authors may consider the use of MG132 in their degradation assays to further corroborate the suggested conclusion of proteasomal degradation of NCA1.
- Moving the paragraph "Lacking of ZmNCA1 reduces…" before the paragraph "A 4-basepair deletion…" may enhance the logical flow of the ms.

Reviewer #2 (Remarks to the Author):

Cao et al
Natural variation of an Ef-hand Ca binding protein ….

This is an interesting paper that talks about saline-alkaline tolerance and through GWAS analysis identifies ZmNCA1. This type of work is interesting basic biology and has links to production agriculture. The fact that this work is done in Maize is a big plus.

I am confused about what is known about this gene outside of maize? The authors should make this clear.

The group goes on to show there is a 4bp deletion in the gene in some varieties that alters translation efficiency and Na tolerance. They show Ca binding via the EF hand – this portion of the work is rather generic and the use of La alone is not that conclusive. They then go on to show a link to transcriptional regulation of PM H+-ATPase. The English in the manuscript could use polishing.

There is a lot of work here including KO and OE studies to confirm the GWAS approach. They have assayed more than 150 lines for variations in ZMNCA1.

Given that this paper is ultimately centered around Na tolerance in alkaline conditions, it would be informative to measure H+-ATPase activity rather than just transcript. Assays of AVP, V-ATPase and SOS1 activity may also be needed- is this regulation specific to the PM H+-ATPase?—I doubt it. I guess this gene regulates several important sodium genes not just one.

The identification portion of this paper is very strong and certainly interesting- the functional characterization phase of the paper is less robust. There has to be better ways to show this is a Ca sensor under these conditions.

Reviewer #3 (Remarks to the Author):

This study has examined a member of the EF-hand Ca binding protein family, named here as ZmNCA1, as a key player in saline-alkaline tolerance in maize, in an attempt to answer an important question for how plants sense saline-alkaline stress and then how this is linked to downstream responses. Furthermore, they address a specific and also important question of how the plasma membrane H+-ATPase is regulated under saline-alkaline conditions. This work has been performed in the agronomically important and increasingly genetically tractable maize species, but which is also salt sensitive.

There is some nice work performed in this study and interesting conclusions are made that are in most parts well supported by the presented data. There is appropriate replication and statistical analysis performed.

There are a number of grammatical and language errors in the manuscript that will need correcting, but this did not restrict the understanding of the work.

There are genes already names NCA1 in Arabidopsis and rice (No Catalase Activity 1) – this does not prevent the authors from referring to their gene as ZmNCA1, but they may wish to consider this in order to avoid potential for confusion. Related to this point, how does ZmNCA1 relate to other EF hand

type proteins? E.g. is it a calmodulin-like family protein (if so consider naming the gene within the CML nomenclature)? Provide some information e.g. a phylogenetic analysis as supplementary data to provide some more information about this gene.

It would be useful to determine further whether the lack of NCA expression (and increased NCA expression) reduces (or increases) root Na content by altered Na uptake, such as via a non-selective cation channel or altered Na efflux across the plasma membrane, such as via the SOS1 Na+/H+ exchanger – as your model suggests? There are chemical inhibitors/blockers (e.g. amiloride for Na+/H+ exchanger, TEA, verapamil, lanthanides, Ca2+, etc that may be used for different types of NSCCs) that could be evaluated to determine the mechanism with better clarity. Alternatively, genetic approaches could be used.

The MHA increased transcript abundance data is convincing but it is unclear whether this is a direct or indirect response to loss of NCA expression. If a direct response, what is the potential mechanism? As yet there is no data to indicate a direct link here so authors must be cautious with their conclusion.

Minor comments.

Please justify the choice of 100 mM concentration of NaHCO3 or NaCl and the pH conditions chosen with regard to agronomic relevance.

On Fig. 1f is the SNP Chr2:12130275 highlighted in red with a different SNP in gray just below (if so what is this SNP?), or is the red spot above the position of the gray SNP simply to indicate that ZmNCA1 is associated with the Chr2:12130275 SNP? Please clarify.

Re: Decision on manuscript NCOMMS-19-18400

Dear Editor,

We are most grateful to you for your invitation of revising and resubmitting our manuscript for publication in Nature Communications. We now have pleasure in submitting a revised manuscript that addresses the points raised by the reviewers.

As reviewer #3 noticed that there were genes already names *NCA1* in Arabidopsis and rice (*No Catalase Activity 1*), **we have renamed *ZmNCA1* into *ZmNSA1* (*Na$^+$ Content under Saline-Alkaline Condition*)** in the revised manuscript, and the following outlines our specific responses to the reviewers' comments:

Reviewer: 1

Review for Manuscript NCOMMS-19-18400

In this submission Cao et al. describe their identification and functional characterization of the EF-hand containing Ca^{2+}-binding protein ZmNCA1. The authors pursued an extensive GWAS-approach encompassing 419 maize inbred lines to identify genetic loci that specifically determine maize tolerance towards saline-alkaline stress. The association analysis identified a SNP in the 3' untranslated region of the NCA1 gene as candidate locus for affecting saline-alkaline tolerance. The authors, moreover, isolated a transposon-induced loss-of-function allele and generated overexpression lines. The phenotype analyses of these plants unambiguously established NCA1 as the locus important for saline-alkaline tolerance and provided evidence that the encoded protein modulates root Na^+ uptake under stress conditions. Subsequently, the authors characterized mechanistic aspects of this function. To this end the authors used an elegant approach of transient protoplast transfection that revealed a role of the SNP in the 3' untranslated region in regulating translation efficiency of NCA1. Moreover, the authors provide experimental evidence that NCA1 is indeed a Ca^{2+} binding protein and that binding of Ca^{2+} appears to result in NCA1 degradation, a finding that is in line with the occurrence of salt stress induced Ca^{2+} signals in plants. Finally, the authors report that degradation of NCA1 confers transcriptional upregulation of specific plasma membrane ATPases. These findings allow the authors to propose a model (which unfortunately is not illustrated by a figure) in which saline-alkaline stress would trigger Ca^{2+} signals, leading to NCA1 degradation, allowing for enhanced root H^+ efflux, counteracting this stress. The authors pursued all technically feasible approaches to illuminate further mechanistic details how NCA1 degradation impacts on ATPase activity (like direct protein interaction) and could finally provide a correlation between the transcriptional regulation of ATPases and NCA1 function. Overall, the findings reported in this work are of greatest general relevance and clearly deserve reporting in a journal addressing a broad audience. The experiments are generally well executed and appropriate controls have been performed.

Our response: We appreciated the reviewer for the positive evaluation of our observations. In order to address the comment "*These findings allow the authors to propose a model (which unfortunately is not illustrated by a figure)……*", we have added a working model of ZmNSA1 in the revised manuscript (**Fig. 8**), *i.e.* under saline-alkaline treatment, the concentration of cytosolic Ca^{2+} increase, Ca^{2+} binds to ZmNSA1 and triggers its degradation via the 26S proteasome pathway, then increases the transcript levels of maize PM H^+-ATPases (*MHA2* & *MHA4*) thus promotes root H^+ efflux, thereby enhancing SOS1 Na^+/H^+ antiporter mediated root Na^+ efflux, ultimately promoting saline-alkaline tolerance.

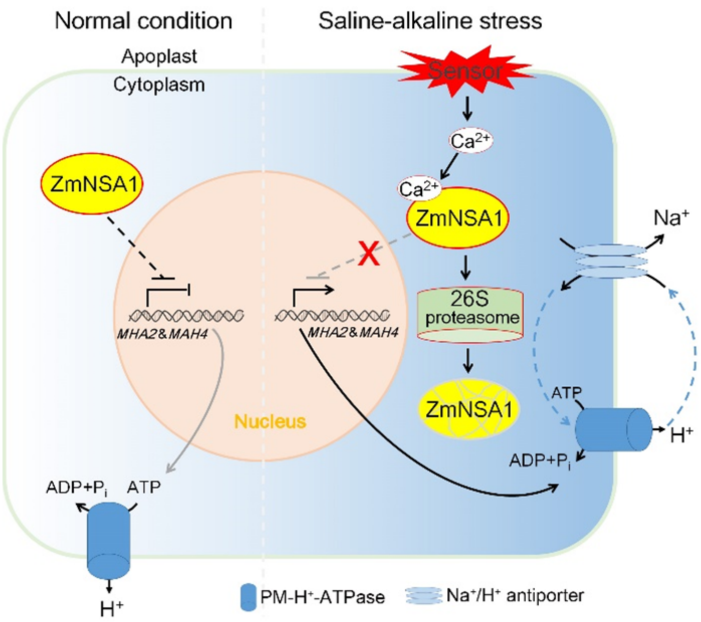

Fig. 8 The working model of ZmNSA1-mediated regulation of Na^+ homeostasis.

Suggestion for writing:

However, the current shape of the manuscript prevents publication. The manuscript appears to be hastily written and is full of mistakes with regards to grammar and writing. Several formulations are awkward to an extent that they interfere with the comprehensibility of the statements. The authors even confused "protoplasts" with "chloroplasts" and "nitrate" with "nitrite".

Our response: We appreciated the reviewer for the valuable comments. We have carefully looked through the manuscript for grammar mistakes and writing.

Further specific comments:

1. The NCA1 protein is not well described. The molecular structure is only rudimentary explained. Moreover, it remains unclear if this protein belongs to a gene family and if related proteins exist in other plant species and if anything is known about the function of related proteins.

Our response: We agree with the reviewer. In order to address this comment, we have conducted further analysis (e.g. phylogenetic analysis), and have added "*ZmNSA1* encodes a Ca^{2+} binding protein, which contains a single EF-hand domain, but with no other domains of known function (**Supplementary Fig. 4**). The orthologs of ZmNSA1 were identified in most plant species (**Supplementary Fig. 5**), but their function remains unknown. Further phylogenetic analysis indicated that ZmNSA1 and its othologs likely have evolutionary relationship with CML family protein (**Supplementary Fig. 6**), however, they haven't been classified as CML family protein in previous study (Mohanta et al., BMC Plant Biology, 2017, 17:38.)" in the Results Section of the revised manuscript.

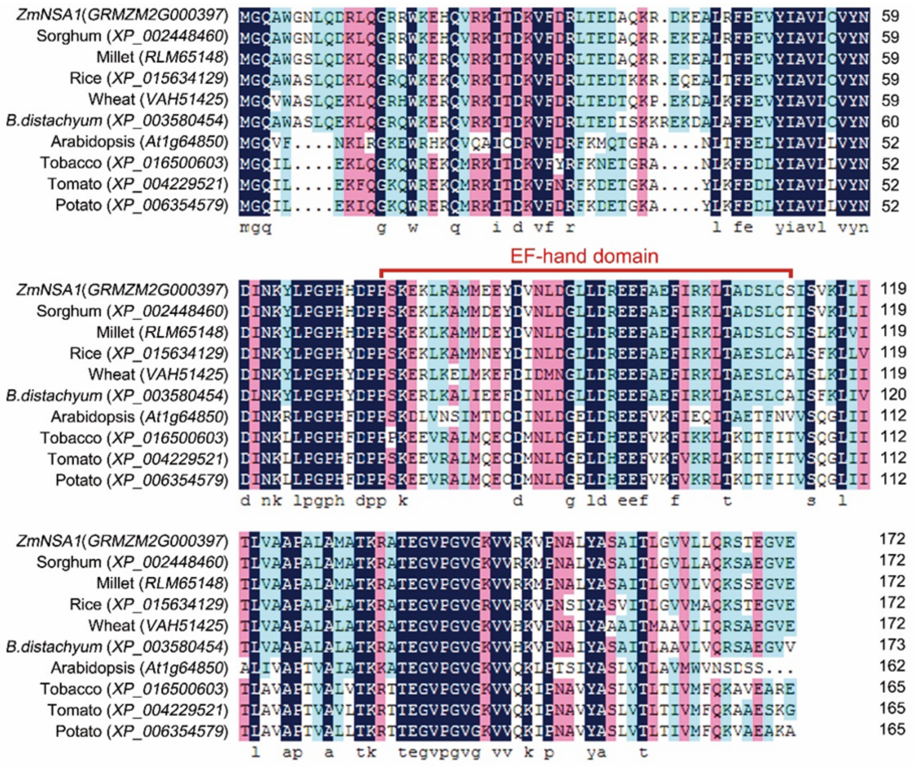

Supplementary Fig. 5 Multiple sequence alignment of ZmNSA1 protein and its orthologs from other selected species.

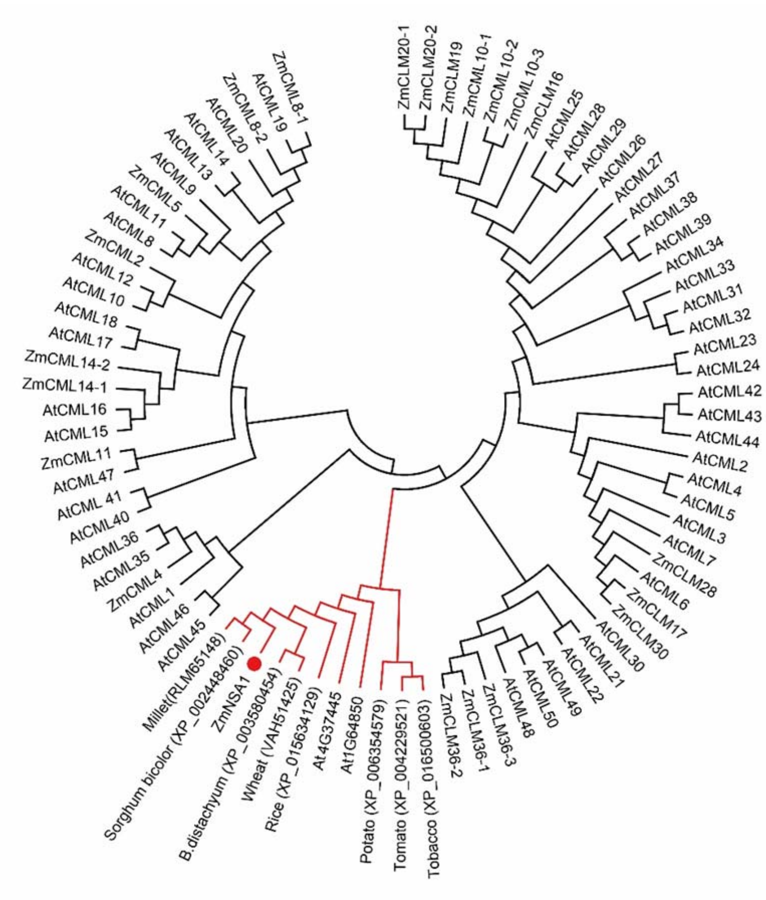

Supplementary Fig. 6 Phylogenetic tree composed of ZmNSA1, selected ZmNSA1 othologs, and CML proteins from maize and Arabidopsis. The CML proteins in maize and Arabidopsis were as described in previous study (Mohanta et al. BMC Plant Biol, 2017, **17**:38.). The red dot highlighted ZmNSA1, and the red lines highlighted the clade contains ZmNSA1 and its orthologs.

2. The authors do not discuss if their determined Ca^{2+} binding properties of NCA1 would be in a range that could be physiologically relevant.

Our response: Thanks for the comment. In order to address the comment, we have added "While previous studies have shown that, in a typical plant cell, free cytoplasmic Ca^{2+} concentrations are in the range of 100 - 200 nM, and increase to 500 - 1000 nM following the onset of external stimulations (e.g. salt stress) (Bose et al., Frontiers in Plant Science, doi: 10.3389/fpls.2011.00085), we observed that the Ca^{2+}-ZmNSA1 binding increase linearly as Ca^{2+} concentrations increase from 100 to 1000 nM (**Figure 5i**), indicating that the Ca^{2+}-ZmNSA1 binding is physiologically relevant." in the revised manuscript.

3. The localization data presented in Fig. 5A may contain artefacts, since for example CBL1-OFP appears to decorate chloroplasts. This is likely a consequence of inappropriate microscopic analyses.

Our response: Thanks for the comment. In order the address the comment, we have replaced **Fig. 5a** with new photos, in which the autofluorescence of chloroplasts were barely detected, and the cytosol-localized pattern of ZmNSA1 is still apparent. In addition, we co-transformed maize protoplasts with ZmNSA1-GFP and Wrky40-mCherry (a nuclear localized protein), and the results showed that ZmNSA1-GFP signal was hardly detected in nuclear (see **Supplementary Fig. 10**).

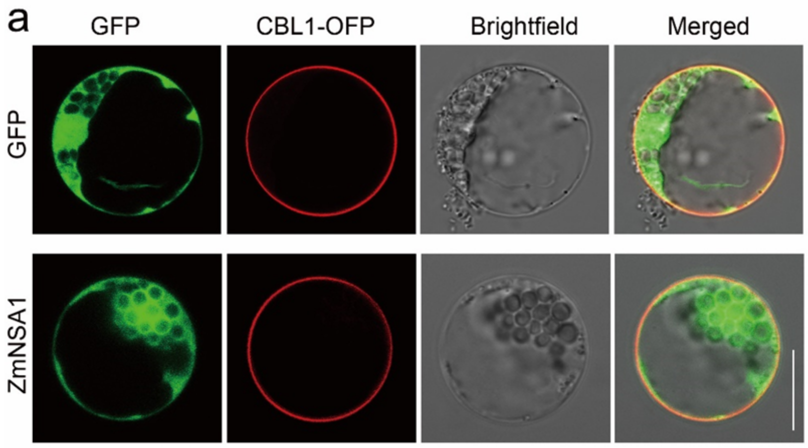

Fig. 5a Subcellular localization of *ZmNSA1*-GFP in maize protoplasts.

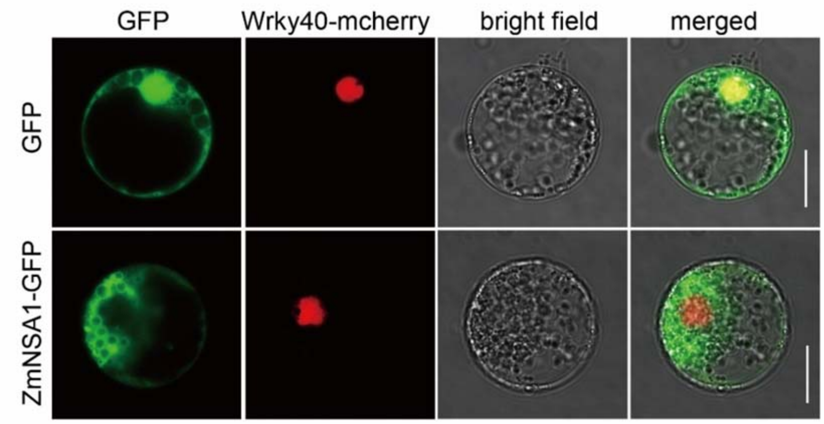

Supplemental Fig. S10 Subcellular localization of *ZmNSA1*-GFP in maize protoplasts.

4. The BiFC analyses (Supplemental Fig. 7) lack expression controls.

Our response: We agree with the reviewer, and we have conducted experiments to analyze the gene expressions in each of the BiFC assays, and the data have been added in the revised manuscript (**Supplemental Fig. 15c-f**).

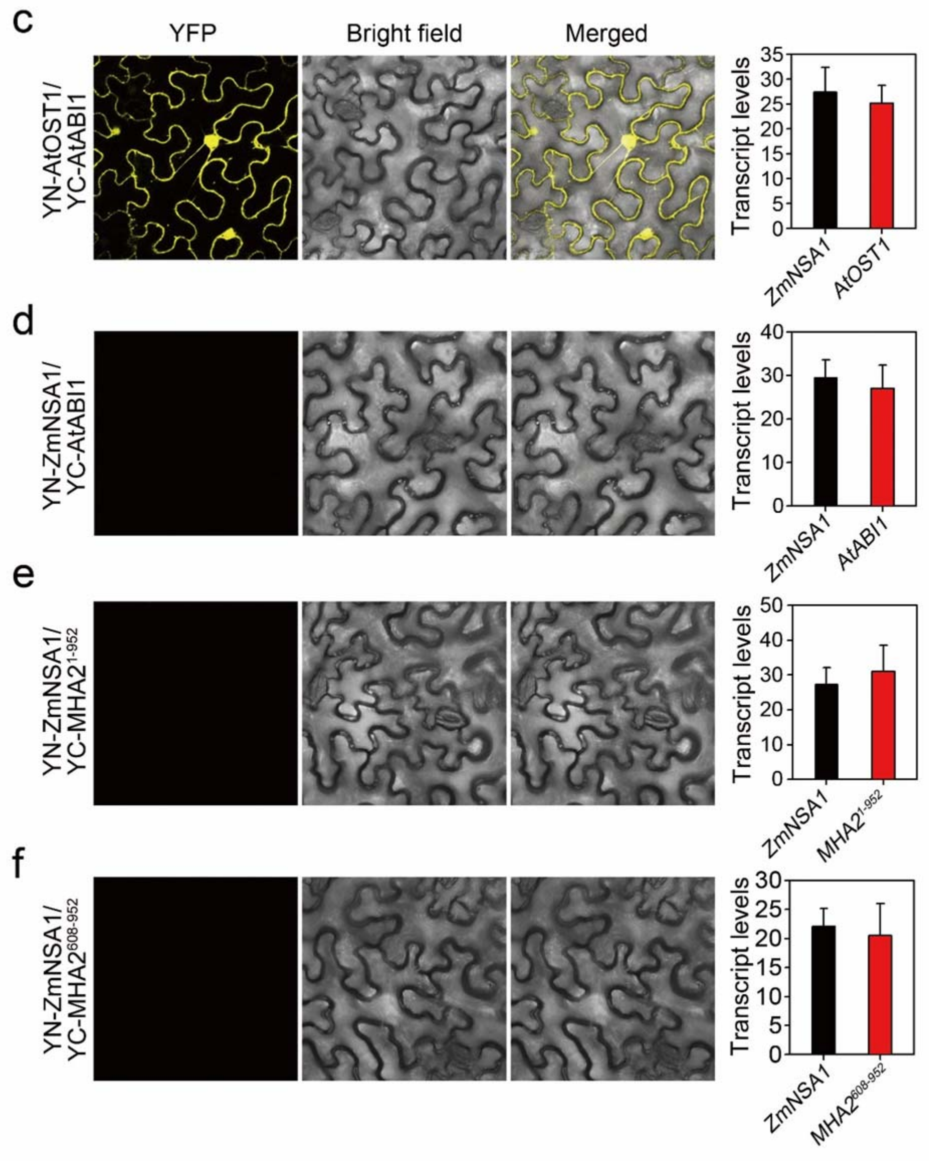

Supplemental Fig. 15 c-f BiFC assay didn't detect the interaction between ZmNSA1 and MHA2 in *N. benthamiana*. The YFP fluorescence was detected by confocal microscopy two days post-infiltration. The transcript levels were expressed as transcript level of indicated genes relative to *ACTIN*. YN-AtOST1/YC-AtABI1 and YN-ZmNSA1/YC-AtBAI1 provided positive and negative controls respectively.

5. The authors used a concentration of 5 mM $LaCl_3$ to induce degradation of NCA1. Although reference concentrations for the application of La^{3+} in maize are currently not available, such a concentration usually kills other plants. Therefore, the authors need to include controls that verify the viability of the La^{3+} treated plants.

Our response: Thanks for the valuable comment. The reference concentrations for the application of La^{3+} in maize are currently not available. As few previous studies used 5 mM La^{3+} for the short-time treatment (e.g. 2h) in Arabidopsis (Li et al., Nature Communications, 2019, doi.org/10.1038/s41467-019-08575-6; Sang et al., Cell Research, 2018, 18:577; Riveras et al., Plant Physiology, 2015, 166:1397), we therefore used 5 mM La^{3+} in our study. In order to address the comment that "*……need to include controls that verify the viability of the La^{3+} treated plants",* we have conducted trypan blue staining to determine if 5 mM La^{3+} treatment for 3 hours kills maize root cells, and we haven't detected apparent cell death in the La^{3+}-treated roots (**Supplementary Fig. 12**).

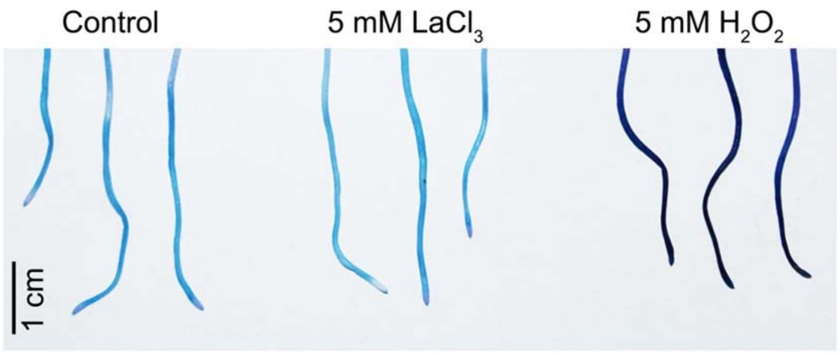

Supplementary Fig. 12 Trypan blue staining of roots that have been treated with 5 mM $LaCl_3$ for 3 hours. The staining of the roots treated with water (Control) and 5 mM H_2O_2 provided negative and positive controls of cell death.

6. On the other hand it would strengthen the conclusiveness of this assay, if the authors could provide any evidence that their La^{3+} treatment indeed inhibits Ca^{2+} dependent processes of salt tolerance (like for example SOS1 activity).

Our response: Thanks for the comment. In order to address the comment, we have analyzed the effect of $LaCl_3$ on root Na^+ efflux , a process substantially mediated by SOS1 Na^+/H^+ antiporter (Yang & Guo, New Phytologist, 2018, **217**: 523.). We observed that, while LaCl3

treatment inhibited saline-alkaline treatment induced degradation of ZmNSA1 (**Fig. 5g**), the treatment reduced root Na^+ efflux (**Supplementary Fig. 13**), which is consistent with the observations that ZmNSA1-overexpressing plants conferred decreased root Na^+ efflux and SOS1 activity (**Fig. 5f, l**).

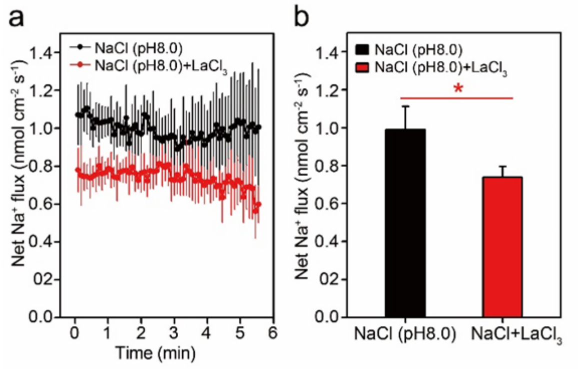

Supplementary Fig. 13 a-b Na^+ flux at the root meristem zone of maize plants with indicated treatments. Five-day-old plants were treated with 100 mM NaCl (pH8.0) for 24h, then treated with $LaCl_3$ for 30 minutes, and then the Na^+ flux were measured using Non-invasive Micro-test Technology (NMT) (see Materials and methods). Data were means ± s.d. $n = 5$. "*" indicated $P < 0.05$.

7. The authors may consider the use of MG132 in their degradation assays to further corroborate the suggested conclusion of proteasomal degradation of NCA1.

Our response: We agree with the reviewer, and have determined the effect of MG132 on the degradation of ZmNSA1. The results indicated that the application of MG132 inhibited ZmNSA1 degradation induced by $NaHCO_3$ treatment, suggesting that ZmNSA1 degradation is depend upon the 26S proteasome pathway. The data have been added in the revised manuscript (**Fig. 5f**).

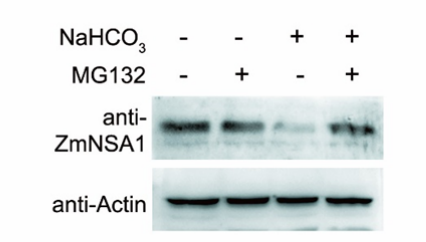

Fig. 5f The influences of MG132 (50 µM) application on ZmNSA1 protein level (the treatments as indicated).

8. Moving the paragraph "Lacking of ZmNCA1 reduces…" before the paragraph "A 4-basepair deletion…" may enhance the logical flow of the ms.

Our response: We thank the reviewer for the kind suggestion, and have thought about the suggestion carefully. Nevertheless, given the paragraph "Lacking of ZmNSA1 reduces…(**Fig. 4**)" is related to the functional characterization of ZmNSA1, we prefer to introduce the natural variation of ZmNSA1 first (the paragraph "A 4-basepair deletion…(**Fig. 3**)"), then introduce **Fig. 4-7**, which together explains the possible mechanism of ZmNSA1-mediated Na^+ homeostasis.

Reviewer #2 (Remarks to the Author):

Cao et al Natural variation of an Ef-hand Ca binding protein …..This is an interesting paper that talks about saline-alkaline tolerance and through GWAS analysis identifies ZmNCA1. This type of work is interesting basic biology and has links to production agriculture. The fact that this work is done in Maize is a big plus.

Our response: We appreciated the reviewer for the positive evaluation of our observations.

Comments to the author:

1. I am confused about what is known about this gene outside of maize? The authors should make this clear.

Our response: Thanks for the comment. A similar comment has also been raised by other reviewer. Please see our response to the Comment #1 of Reviewer #1.

2. They show Ca binding via the EF hand – this portion of the work is rather generic and the use of La alone is not that conclusive.

Our response: We thank the reviewer for the comment. It is probably not surprising that we showed Ca^{2+} binds to the EF hand domain of ZmNSA1, nevertheless, it is novel to observe that such a binding triggers the degradation of ZmNSA1, then increases the transcription of PM H^+-ATPases. In order to address the comment "*the use of La alone is not that conclusive*", we have determined the effect of verapamil (another Ca^{2+} channel blocker) on the degradation of ZmNSA1, and the result indicated that the application of verapamil also inhibited $NaHCO_3$-induced degradation of ZmNSA1, supporting our conclusion that the $NaHCO_3$

induces the degradation of ZmNSA1 by a Ca^{2+} dependent manor (**Supplementary Fig. 11**). In addition, with the suggestion from Reviewer #1, we have also found that $NaHCO_3$ induced degradation of ZmNSA1 via 26S proteasome pathway (**Fig. 5f**).

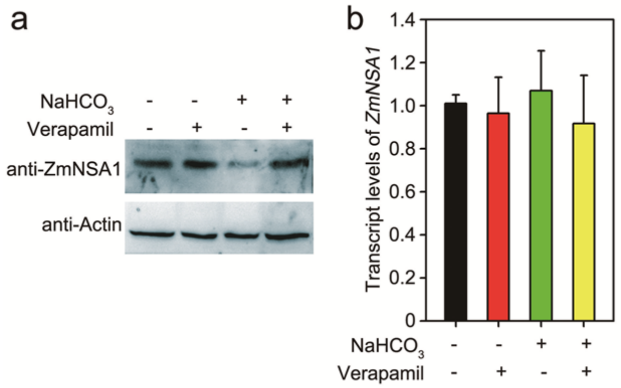

Supplementary Fig. 11 The influences of verapamil (100 μM) application on *ZmNSA1* protein (**a**) and transcript (**b**) levels (genotypes and treatments as indicated). Data represent three replicates, and data in **b** were means ± s.d. of three replicates.

3. The English in the manuscript could use polishing.

Our response: We appreciated the reviewer for the valuable comments. We have carefully looked through the manuscript to improve the English.

4. Given that this paper is ultimately centered around Na tolerance in alkaline conditions, it would be informative to measure H^+-ATPase activity rather than just transcript. Assays of AVP, V-ATPase and SOS1 activity may also be needed- is this regulation specific to the PM H^+-ATPase?—I doubt it. I guess this gene regulates several important sodium genes not just one.

Our response: Thanks for the valuable comment. We have measured the PM H^+-ATPase and SOS1 Na^+/H^+ antiporter activities in *ZmNSA1*^{UFMu} and *ZmNSA1*-overexpressing lines, using the methods described in previous studies (Qiu et al., PNAS, 2002, 99:8436; Yang et al., Plant Cell 2010, 22:1313). The results indicated that *ZmNSA1*^{UFMu} conferred greater PM H^+-ATPase (**Fig. 6g, h**) and SOS1 Na^+/H^+ antiporter activities than W22 (**Fig. 4k**), and *ZmNSA1*-overexpressing plants conferred lower PM H^+-ATPase (**Fig. 6i, j**) and SOS1 Na^+/H^+ antiporter activities than wild type (**Fig. 4l**). In addition, we have also measured the V-H^+-ATPase and V-H^+-PPase activities in *ZmNSA1*^{UFMu}, using the methods described in

previous studies (Queirós et al., Journal of Experimental Botany 2009, 60:1363), and found there were no detectable differences between *ZmNSA1*^{UFMu} and W22 (**Supplementary Fig. 14**). These results support our conclusion that ZmNSA1 regulates PM H^+-ATPase mediated root H^+ efflux, thereby influences the activity of SOS1 Na^+/H^+ antiporter. These data also suggest that ZmNSA1 has minimal effect on the activities of V-H^+-ATPase and V-H^+-PPase, Nevertheless, we agree with the reviewer that it remains possible that *ZmNSA1* might regulate other untested factors associated with Na^+ homeostasis as well.

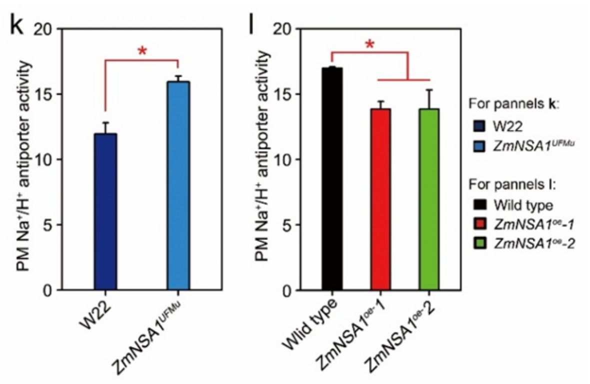

Fig. 4 k-l. The activity of SOS1 Na^+/H^+ antiporter in the plasma membrane vesicles isolated from the roots of $NaHCO_3$ treated plants (genotypes as indicated). Transport assays were performed as described in Materials and Methods. The data showed the calculated activity of SOS1 Na^+/H^+ antiporter (**k**, **l**). Data were means ± s.d. of three replicates. "*" indicated $P < 0.05$.

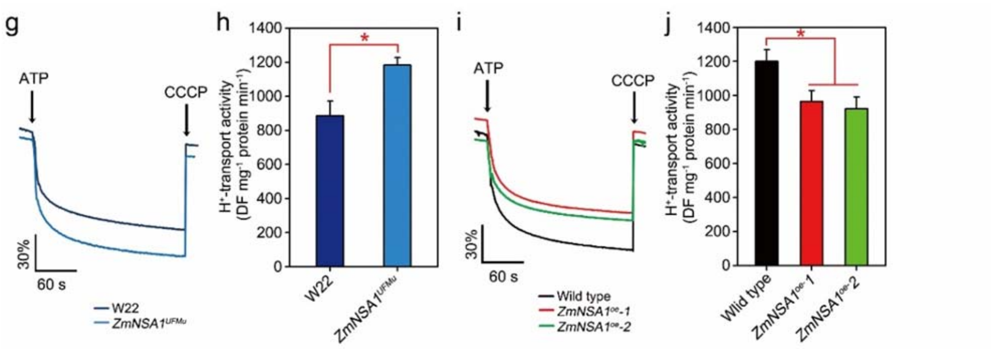

Fig. 6g-j. The activity of PM-H^+-ATPase in plasma membrane vesicles isolated from the roots of $NaHCO_3$ treated plants (genotypes as indicated). Transport assays were performed as described in Materials and Methods. The data showed the timely varying curves of quinacrine fluorescent intensity (**g**, **i**) and the calculated activity of PM-H^+-ATPase (**h**, **j**). Data were means ± s.d. of three replicates. "*" indicated $P < 0.05$.

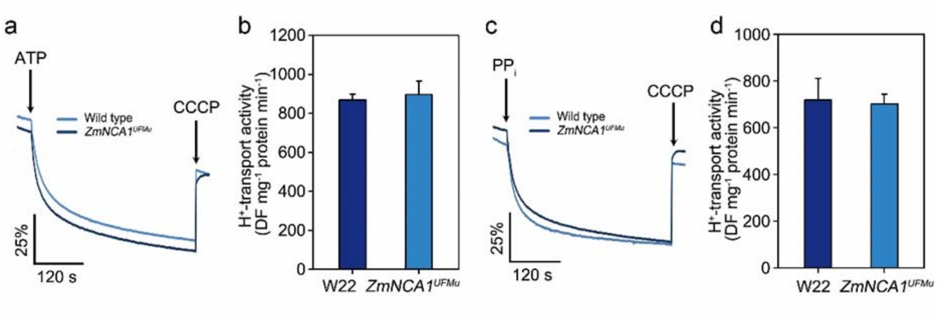

Supplementary Fig. 14 The activity of V-H^+-ATPase and V-H^+-PPase in the tonoplast vesicles isolated from roots of $NaHCO_3$ treated plants (genotypes as indicated). Timely varying curves of quinacrine fluorescent intensity (**a**, **c**) and the calculated activity of V-H^+-ATPase (**b**) and V-H^+-PPase (**d**). Data were means ± s.d. of three replicates. "*" indicated $P < 0.05$.

5. The identification portion of this paper is very strong and certainly interesting- the functional characterization phase of the paper is less robust. There has to be better ways to show this is a Ca sensor under these conditions.

Our response: We thank the reviewer for the positive evaluation of our identification of ZmNSA1, and we agree with the reviewer that the functional characterization phase of the paper is less robust. With the suggestions from all reviewers, we have conducted further experiments to strength the mechanism of ZmNSA1-mediated regulation of Na^+ homeostasis, including: (1) new data indicated that MG132 can inhibit $NaHCO_3$ induced degradation of ZmNSA1 (**Fig. 5f**), which advances our mechanistic understanding of Ca^{2+} triggered degradation of ZmNSA1; (2) we have analyzed the activities of PM-H^+-ATPase and SOS1 Na^+/H^+ antiporter (**Fig. 6g-j** and **Fig. 4k, l**), the results strengthened our conclusion that ZmNSA1 regulates root H^+ efflux, thereby influencing SOS1 Na^+/H^+ antiporter-mediated Na^+ efflux; (3) we have proposed a working model of ZmNSA1-mediated Na^+ homeostasis under saline-alkaline condition (**Fig. 8**), *i.e.* under saline-alkaline treatment, the concentration of cytosolic Ca^{2+} increase, Ca^{2+} binds to ZmNSA1 and triggers its degradation via the 26S proteasome pathway, then increases the transcript levels of maize PM H^+-ATPases (*MHA2* & *MHA4*) thus promotes root H^+ efflux, thereby enhancing SOS1 Na^+/H^+ antiporter mediated root Na^+ efflux, ultimately promoting saline-alkaline tolerance. We hope these revisions can satisfy the comments "*the functional characterization phase of the paper is less robust*".

We have added "While previous studies have shown that, in a typical plant cell, free cytoplasmic Ca^{2+} concentrations are in the range of 100 - 200 nM, and increase to 500 - 1000 nM following the onset of external stimulations (e.g. salt stress) (Bose et al., Frontiers in Plant Science, 2011, doi:10.3389/fpls.2011.00085), we observed that the Ca^{2+}-ZmNSA1 binding increase linearly as Ca^{2+} concentrations increase from 100 to 1000 nM (**Figure 5i**), indicating that the Ca^{2+}-ZmNSA1 binding is physiologically relevant." in the revised manuscript. This discussion highlighted that ZmNSA1 binds Ca^{2+} at physiologically relevant concentration, thus ideally could involve in the decoding of Ca^{2+} signature under saline-alkaline conditions. We hope the revision can to some extend satisfy the comment *"there has to be better ways to show this is a Ca^{2+} sensor under these conditions"*.

Reviewer #3 (Remarks to the Author):

This study has examined a member of the EF-hand Ca binding protein family, named here as ZmNCA1, as a key player in saline-alkaline tolerance in maize, in an attempt to answer an important question for how plants sense saline-alkaline stress and then how this is linked to downstream responses. Furthermore, they address a specific and also important question of how the plasma membrane H^+-ATPase is regulated under saline-alkaline conditions. This work has been performed in the agronomically important and increasingly genetically tractable maize species, but which is also salt sensitive.

There is some nice work performed in this study and interesting conclusions are made that are in most parts well supported by the presented data. There is appropriate replication and statistical analysis performed.

Our response: We appreciated the reviewer for the positive evaluation of our observations.

Major comments:

1. There are a number of grammatical and language errors in the manuscript that will need correcting, but this did not restrict the understanding of the work.

Our response: We appreciated the reviewer for the valuable comments. We have carefully looked through the manuscript for grammatical and language errors.

2. There are genes already names NCA1 in Arabidopsis and rice (No Catalase Activity 1) – this does not prevent the authors from referring to their gene as ZmNCA1, but they may wish to consider this in order to avoid potential for confusion. Related to this point, how does

ZmNCA1 relate to other EF hand type proteins? E.g. is it a calmodulin-like family protein (if so consider naming the gene within the CML nomenclature)? Provide some information e.g. a phylogenetic analysis as supplementary data to provide some more information about this gene.

Our response: We thank the reviewer for noticing that there were genes already names *NCA1* in Arabidopsis and rice. We have renamed our gene into *ZmNSA1* (*Na$^+$ Content under Saline-Alkaline Condition*) in the revised manuscript. We also thank the reviewer for the comments "*how does ZmNSA1 relate to other EF hand type proteins?*" and "*Provide some information e.g. a phylogenetic analysis as supplementary data to provide some more information about this gene*". Similar comments have been raised by other reviewer, and have been addressed substantially in the revised manuscript. Please see our response to Comment #1 of Reviewer #1.

3. It would be useful to determine further whether the lack of NCA expression (and increased NCA expression) reduces (or increases) root Na content by altered Na uptake, such as via a non-selective cation channel or altered Na efflux across the plasma membrane, such as via the SOS1 Na^+/H^+ exchanger – as your model suggests? There are chemical inhibitors/blockers (e.g. amiloride for Na^+/H^+ exchanger, TEA, verapamil, lanthanides, Ca^{2+}, etc that may be used for different types of NSCCs) that could be evaluated to determine the mechanism with better clarity. Alternatively, genetic approaches could be used.

Our response: Thanks for the valuable comment. In order the address the comment, firstly, we have determined the effect of the Na^+/H^+ antiporter inhibitor amiloride on root Na^+ efflux of *ZmNSA1^{UFMu}* and *ZmNSA1*-overexpressing plants. The results indicated that, while *ZmNSA1^{UFMu}* conffered greater root Na^+ efflux and *ZmNSA1*-overexpressing plants conferred lower root Na^+ efflux than wild type (**Fig. 4e, f**), the application of amiloride reduceed root Na^+ efflux of all genotypes, but with different degrees of reduction (**Fig. 4g-j)**. Consequently, amiloride application reduced the differences of root Na^+ efflux between *ZmNSA1^{UFMu}* and wild type (W22) (**Fig. 4g, h**), and between *ZmNSA1*-overexpressing plants and wild type (**Fig. 4i, j**), suggesting that *ZmNSA1*-mediated regulation of root Na^+ efflux is of Na^+/H^+ antiporter dependent. Secondly, we have measured the activity of SOS1 Na^+/H^+ antiporter, and observed that *ZmNSA1^{UFMu}* conferred greater SOS1 Na^+/H^+ antiporter activity (**Fig. 4k**) than wild type,

and *ZmNSA1*-overexpressing plants conferred lower SOS1 Na^+/H^+ antiporter activity than wild type (**Fig. 4l**). These data support our model that ZmNSA1 regulates root H^+ efflux, then influences SOS1 Na^+/H^+ antiporter mediated root Na^+ efflux (**Fig. 8**).

We agree with the comment that ZmNAS1 may also involves in the regulation of Na^+ uptake. We have accordingly in recent weeks tried to use Non-invasive Micro-test Technology (NMT) to determine the possibility, as described in previously study (Chakraborty et al., Journal of Experimental Botany, 2016, 67:4611). Nevertheless, we found that NMT assay of maize roots yield massive fluctuation of signal when the recording buffer contains high concentration of Na^+ (e.g. 100 mM) (see the underneath **Supporting Figure for Comment #3 of Reviewer #3**), which then not permit us to carry out further experiment with this method (e.g. test the effect of the chemical inhibitors on the Na^+ uptake). We then choose an alternative approach to determine if ZmNAS1 regulates root Na^+ uptake, i.e. by measuring the root Na^+ contents in the roots of the plants that have been treated with saline-alkaline stress for short time (10 minutes), such experiment could to some extent reflect the short-term Na^+ uptake by roots. We found that the Na^+ contents in the treated roots of *ZmNSA1^UFMu^* and *ZmNSA1*-overexpressing plants were comparable with that of wild type controls, suggesting that ZmNAS1 is unlikely associated with the regulation of Na^+ uptake (**Supplementary Fig. 9**).

Taken together, the revised manuscript has substantially strengthen our conclusion that ZmNAS1 involves in the regulation of root Na^+ efflux, but is unlikely associated with the regulation of root Na^+ uptake.

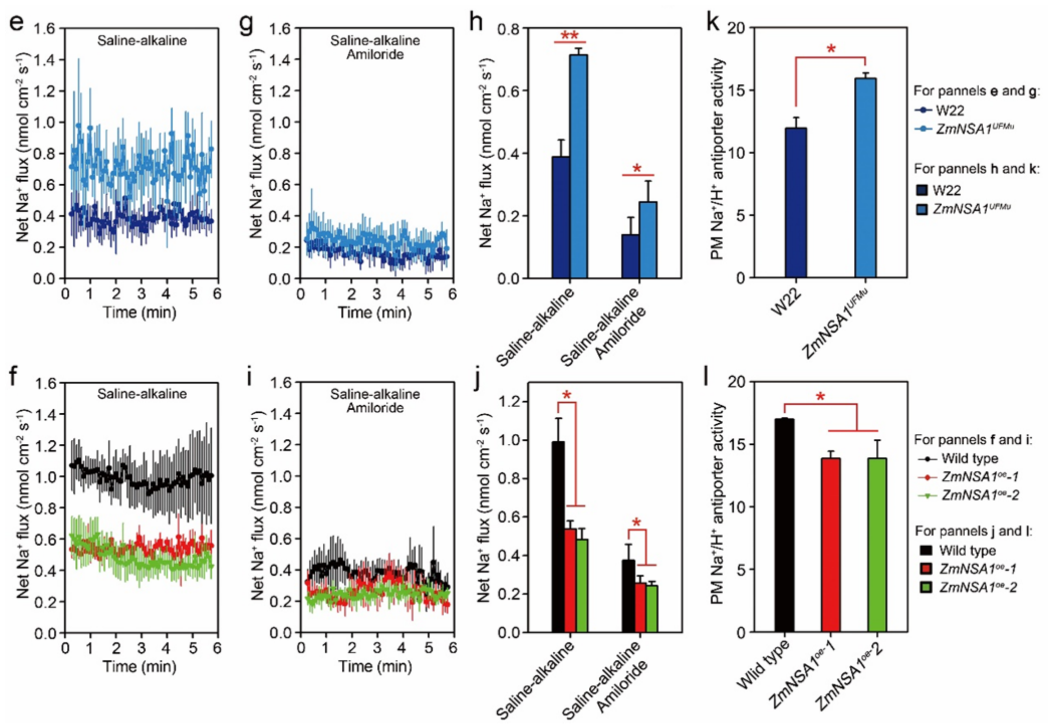

Fig 4. e-h Na^+ flux at the root meristem zone of *ZmNSA1^{UFMu}* and W22 plants. Five-day-old plants were treated with 100 mM NaCl (pH8.0) for 24h, incubate in recording buffer (**e**) or recording buffer with 50 μM amiloride (**f**) for 30 mins, then the Na^+ flux were measured using Non-invasive Micro-test Technology (NMT) (see Materials and methods). ……**f-j** Na^+ flux at the meristem zone of wild type and *ZmNSA1*-overexpressing plants (treatments as indicated). **k**, **l** The activity of SOS1 Na^+/H^+ antiporter in plasma membrane vesicles isolated from the roots of $NaHCO_3$ treated plants (genotypes as indicated). Transport assays were performed as described in Materials and Methods. Data in **e-h** and **f-j** were means ± s.d. $n = 5$. Data in **k** and **l** were means ± s.d. of three replicates. "*" and "**" indicate $P < 0.05$ and $P < 0.01$ respectively.

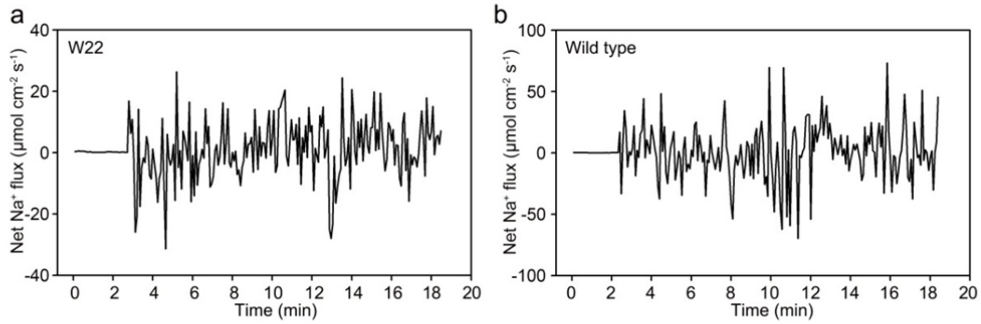

Supporting Figure for Comment #3 of Reviewer #3 Na^+ flux recorded by Non-invasive Micro-test Technology (NMT) in the recording buffer contains 100 mM Na^+. Two representative examples were displayed.

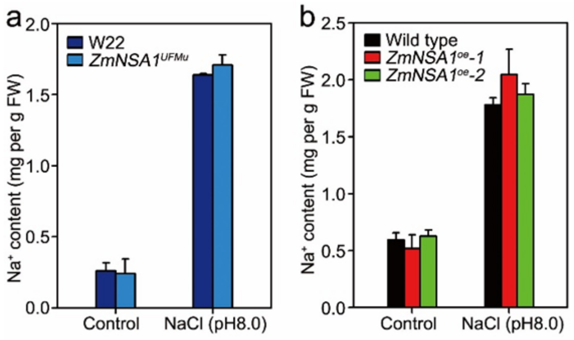

Supplementary Fig. 9 Root Na^+ contents of plants that have been treated with 100 mM NaCl (pH8.0) for 10 minutes.

4. The MHA increased transcript abundance data is convincing but it is unclear whether this is a direct or indirect response to loss of NCA expression. If a direct response, what is the potential mechanism? As yet there is no data to indicate a direct link here so authors must be cautious with their conclusion.

Our response: Thanks for the comment. As mentioned by the reviewer, our results have shown that the Ca^{2+} triggered degradation of ZmNSA1 is clearly associated with the increase of *of MHAs* expression (**Figure 7**). As ZmNSA1 has no transcription activation domain and is barely detected in nucleus (**Supplementary Fig. 10**), the ZmNSA1-mediated regulation of *MHAs* transcription is likely to be an indirect response. We have added such a notion in the discussion section of the revised manuscript.

Minor comments:

1. Please justify the choice of 100 mM concentration of NaHCO3 or NaCl and the pH conditions chosen with regard to agronomic relevance.

Response: Thanks for the comments. In order to address this comment, we added “Given sodium hydrogen carbonate ($NaHCO_3$) is one of the major basic salts in nature environments, we used 100 mM $NaHCO_3$ to mimic the saline-alkaline stress, and both the Na^+ concentration (100 mM) and pH value (pH8.8) were agronomic relevance (Bao et al. Bioengineered, 2016, 7: 372; Munns et al., 2012, Nat Biotechnol, 30: 360).”

2. On Fig. 1f is the SNP Chr2:12130275 highlighted in red with a different SNP in gray just below (if so what is this SNP?), or is the red spot above the position of the gray SNP simply to

indicate that ZmNCA1 is associated with the Chr2:12130275 SNP? Please clarify.

Our response: Thanks for the comment. The gray one below Chr2:12130275 is another SNP (Chr2_12130134). We have clarified this in the revised manuscript, and we have highlighted SNP12130134 in red in the revised manuscript.

REVIEWERS' COMMENTS:

Reviewer #1 (Remarks to the Author):

Review for NCOMMS-19-18400:
The authors have performed an impressive number of additional experiments to address my comments and have also carefully edited the previous version of their manuscript. In consequence, the revised version has fully addressed all my concerns. The included model nicely illustrates the important and novel findings reported by the authors. The available data now fully support the conclusions of this work. I feel that this work now provides very important and novel insights into the role of NCA1 and Ca2+ signaling in conferring tolerance to saline-alkaline conditions in maize that are of interest for a general audience.
Minor comment:
Line 87: I recommend to reformulate "for the first time" into a more elegant formulation to claim priority.

Reviewer #2 (Remarks to the Author):

The authors have done a lot of work here on the revision and have satisfied my previous concerns.

Reviewer #3 (Remarks to the Author):

The authors have been very thorough in addressing all of the comments by all three reviewers. I feel that this is now a very strong study and all of the conclusions made are clearly supported by the provided data. I accept all of the authors responses and rebuttals to my original comments and have no further comments to make based on the science of this study.

There are still a small number of language and grammatical errors in parts of the text that will either need correcting by the authors or correcting during the copy editing process.

Reviewer #4 (Remarks to the Author):

As suggested by the editor, I only look at the GWAS part of the manuscript, which is rather concise. In general I feel the GWAS part looks OK, although more details may be added to ensure the paper to be self-contained. Please find my suggestions below for the authors' consideration.

First, there is no citation regarding which tool is used for linear mixed model where it is mentioned for the first time in the manuscript. Later on, the authors cited TASSEL at the place for sequencing follow up. It is unclear for me whether the authors use another tool to conduct the initial analysis and then use TASSEL for fine mapping, or the entire analyses are carried out using TASSEL only.

Second, the authors cited reference 49 and 51 for the generation of the genotype data. It might be helpful to provide a bit more details, e.g., some summary statistics, on the data generation.

Third, as the minimal requirement in presentations of a GWAS signal, one may provide a full Manhattan plot (instead of only 2.5Mb in Chromosome 2) to show the overall picture of the genetic architecture of the trait. Also, a QQ plot of expected and observed P-values needs to be included to justify that there is no inflation of the distribution of P-values.

Reviewer #1 (Remarks to the Author):

Review for NCOMMS-19-18400:

The authors have performed an impressive number of additional experiments to address my comments and have also carefully edited the previous version of their manuscript. In consequence, the revised version has fully addressed all my concerns. The included model nicely illustrates the important and novel findings reported by the authors. The available data now fully support the conclusions of this work. I feel that this work now provides very important and novel insights into the role of NSA1 and Ca2+ signaling in conferring tolerance to saline-alkaline conditions in maize that are of interest for a general audience.

Our Response: Thanks the reviewer for the positive evaluation of our revised manuscript.

Minor comment:

Line 87: I recommend to reformulate "for the first time" into a more elegant formulation to claim priority.

Our Response: Thanks for the suggestion. We have reformulate the sentence into "Our study shows how……..".

Reviewer #2 (Remarks to the Author):

The authors have done a lot of work here on the revision and have satisfied my previous concerns.

Our Response: Thanks the reviewer for the positive evaluation of our revised manuscript.

Reviewer #3 (Remarks to the Author):

The authors have been very thorough in addressing all of the comments by all three reviewers. I feel that this is now a very strong study and all of the conclusions made are clearly supported by the provided data. I accept all of the authors responses and rebuttals to my original comments and have no further comments to make based on the science of this study. There are still a small number of language and grammatical errors in parts of the text that will either need correcting by the authors or correcting during the copy editing process.

Our Response: We appreciated the reviewer for the positive evaluation. We have carefully looked through the English usage and corrected the grammatical errors.

Reviewer #4 (Remarks to the Author):

As suggested by the editor, I only look at the GWAS part of the manuscript, which is rather concise. In general I feel the GWAS part looks OK, although more details may be added to ensure the paper to be self-contained. Please find my suggestions below for the authors' consideration.

First, there is no citation regarding which tool is used for linear mixed model where it is mentioned for the first time in the manuscript. Later on, the authors cited TASSEL at the

place for sequencing follow up. It is unclear for me whether the authors use another tool to conduct the initial analysis and then use TASSEL for fine mapping, or the entire analyses are carried out using TASSEL only.

Our Response: Thanks for the comment. The entire analyses were carried out using TASSEL 3.0, under mixed linear model (MLM). We have made this clear in the revised manuscript.

Second, the authors cited reference 49 and 51 for the generation of the genotype data. It might be helpful to provide a bit more details, e.g., some summary statistics, on the data generation.

Our Response: Thanks for the comment. We provided the strategies of generating the genotypes in the revised manuscript, and showed that 56,110 SNPs were detected by Maize SNP50 array, the rest SNPs were detected by RNA-seq in the revised manuscript.

Third, as the minimal requirement in presentations of a GWAS signal, one may provide a full Manhattan plot (instead of only 2.5Mb in Chromosome 2) to show the overall picture of the genetic architecture of the trait. Also, a QQ plot of expected and observed P-values needs to be included to justify that there is no inflation of the distribution of P-values.

Our Response: We appreciated the reviewer for the valuable comments. We provided the full Manhattan plot and QQ plot in the revised manuscript (Supplementary Fig. 3). And the QQ plot showed that there is no obvious inflation of the distribution of *P*-values.
